# Inference in conditioned dynamics through causality restoration

**DOI:** 10.1038/s41598-023-33770-3

**Published:** 2023-05-05

**Authors:** Alfredo Braunstein, Giovanni Catania, Luca Dall’Asta, Matteo Mariani, Anna Paola Muntoni

**Affiliations:** 1 DISAT, Politecnico di Torino, Corso Duca Degli Abruzzi 24, 10129 Turin, Italy; 2 INFN, Sezione di Torino, Turin, Italy; 3 Italian Institute for Genomic Medicine, IRCCS Candiolo, SP-142, 10060 Candiolo, TO Italy; 4 Departamento de Física Téorica I, Universidad Complutense, 28040 Madrid, Spain; 5 Collegio Carlo Alberto, P.za Arbarello 8, 10122 Turin, Italy

**Keywords:** Complex networks, Statistical physics, Biological physics

## Abstract

Estimating observables from conditioned dynamics is typically computationally hard. While obtaining independent samples efficiently from unconditioned dynamics is usually feasible, most of them do not satisfy the imposed conditions and must be discarded. On the other hand, conditioning breaks the causal properties of the dynamics, which ultimately renders the sampling of the conditioned dynamics non-trivial and inefficient. In this work, a Causal Variational Approach is proposed, as an approximate method to generate independent samples from a conditioned distribution. The procedure relies on learning the parameters of a generalized dynamical model that optimally describes the conditioned distribution in a variational sense. The outcome is an effective and unconditioned dynamical model from which one can trivially obtain independent samples, effectively restoring the causality of the conditioned dynamics. The consequences are twofold: the method allows one to efficiently compute observables from the conditioned dynamics by averaging over independent samples; moreover, it provides an effective unconditioned distribution that is easy to interpret. This approximation can be applied virtually to any dynamics. The application of the method to epidemic inference is discussed in detail. The results of direct comparison with state-of-the-art inference methods, including the soft-margin approach and mean-field methods, are promising.

## Introduction

The method we will present is rather general and applies to a wide family of stochastic processes. We will thus first describe it below in complete generality, and delay its description for a specific important application (namely the risk assessment problem in epidemic spreading processes) to the following section.

Let us denote by $\mathbb {P}[\textbf{x}]=\mathbb {P}\left[ {x}\left( 0\right) ,\ldots ,{x}\left( k\Delta t\right) \right]$ the probability distribution of trajectories $\textbf{x}$ of a (known) dynamical model. Given a (hidden) realization $\textbf{x}^{*}$, consider a set of observations $\mathscr {O}=({O}_1,\dots ,{O}_M)$ sampled from a (known) conditional distribution $\mathbb {P}\left[ \mathscr {O}|\textbf{x}^{*}\right]$. The scope of this work is to devise an efficient method to infer information about $\textbf{x}^{*}$ given $\mathscr {O}$, in particular, to be able to estimate averages over the posterior distribution

1 $$\begin{aligned} \mathbb {P}\left[ \textbf{x}|\mathscr {O}\right] =\mathbb {P} [\textbf{x}]\mathbb {P}\left[ \mathscr {O}|\textbf{x}\right] \mathbb {P}\left[ \mathscr {O}\right] ^{-1}. \end{aligned}$$

Although it might be generally feasible to sample efficiently from the prior $\mathbb {P}[\textbf{x}]$, sampling from $\mathbb {P}\left[ \textbf{x}|\mathscr {O}\right]$ is normally difficult. A naive approach is given by importance sampling^1,2^, that consists in evaluating the average of a function *f* by first generating *M* independent samples $\textbf{x}^{1},\dots ,\textbf{x}^{M}$ from $\mathbb {P}[\textbf{x}]$ and then computing

2 $$\begin{aligned} \left\langle f\right\rangle \approx \frac{\sum _{\mu =1}^{M}f\left( \textbf{x}^{\mu }\right) \mathbb {P}\left[ \mathscr {O}|\textbf{x}^{\mu }\right] }{\sum _{\mu =1}^{M} \mathbb {P}\left[ \mathscr {O}|\textbf{x}^{\mu }\right] }. \end{aligned}$$

Unfortunately, this method is impractical when observations deviate significantly from the typical case, as for the case in which $\mathbb {P}\left[ \mathscr {O}|\textbf{x}^{\mu }\right]$ becomes very small (or even zero), rendering the convergence of $\left\langle f\right\rangle$ to the true average value inefficient.

One reason for which sampling from $\mathbb {P}[\textbf{x}]$ is usually feasible is that the causal structure induced by the dynamical nature of the stochastic process can be exploited to efficiently generate trajectories. The *causal* property of the stochastic dynamics lies in the fact that the state of the system at a given time depends naturally (in a stochastic way) on states at previous times. When considering discrete time-steps or epochs $0,\Delta t,2\Delta t,\dots$ (in the following discussion, for simplicity of notation, we will assume $\Delta t=1$), this property implies that the distribution of trajectories of the stochastic dynamics assumes the following factorized form:

3 $$\begin{aligned} \mathbb {P}\left[ {x}(0),\dots ,{x}(T)\right] = \,\prod _{t=0}^{T}\mathbb {P}\left[ {x}(t)|{x}(t-1),\dots ,{x}(0)\right] , \end{aligned}$$

where in the $t=0$ term the conditioning part is empty and thus the probability is unconditioned. In most models, it is computationally simple (or at least feasible) to sample *x*(*t*) from $\mathbb {P}\left[ x(t)|x(t-1),\dots ,x(0)\right]$, implying that (3) can be exploited to generate trajectories by sequentially sampling *x*(0), then *x*(1), etc.

The intrinsic difficulty associated with sampling from the conditioned distribution $\mathbb {P}\left[ \textbf{x}|\mathscr {O}\right]$ is a consequence of *causality breaking*^3^ induced by the addition of the extra information in $\mathscr {O}$. In general, $\mathbb {P}\left[ x(t)|x(t-1),\dots ,x(0)\right]$ and $\mathbb {P}\left[ x(t)|x(t-1),\dots ,x(0),\mathscr {O}\right]$ are very different objects. For example, even if the former is time and space invariant, the latter is generally not, because this symmetry is typically broken by the observations. This difference ultimately implies that we cannot sample from the posterior distribution sequentially as in the unconditioned case (3). Although we can write an exact expression similar to (3),

4 $$\begin{aligned} \mathbb {P}\left[ \textbf{x}|\mathscr {O}\right] =\prod _{t=0}^T \mathbb {P}\left[ {x}(t)|{x}(t-1),\dots ,{x}(0),\mathscr {O}\right] , \end{aligned}$$

sampling from (4) is unfortunately still problematic. Indeed, the expression for $\mathbb {P}\left[ x(t)|x(t-1),\dots ,x(0),\mathscr {O}\right]$ is in general extremely difficult to compute and involves a marginalization over times $t'>t$ (with an exponential number of terms):

5 $$\begin{aligned} \mathbb {P}\left[ x(t)|x(t-1),\dots ,x(0),\mathscr {O}\right]&\propto \sum _{x(t+1),\dots ,x(T)} \prod _{t'=0}^{T}\mathbb {P}\left[ x(t')|x(t'-1),\dots ,x(0)\right] \mathbb {P}\left[ \mathscr {O}|x(T),\dots ,x(0)\right] . \end{aligned}$$

This dependence on future times is in our opinion the real source of the causality breaking phenomenon.

When dynamics are unconditioned, i.e. causality applies, information is intuitively flowing from past to future. Although it is a very intuitive concept, the study of information flow is actually rather involved and it opens to interesting insights into the collective interactions among agents in agent-based systems. We refer the interested reader to^4,5^. The approach proposed here, called *Causal Variational Approach* (CVA), aims at providing a variational approximation of the posterior distribution $\mathbb {P}\left[ \textbf{x}|\mathscr {O}\right]$, for which causality features are restored and, therefore, independent samples can be efficiently generated from it. In particular, we propose to approximate $\mathbb {P}\left[ \textbf{x}|\mathscr {O}\right] \approx Q(\textbf{x})$, where $Q\left( x(0),\dots ,x(T)\right) =\prod _{t=0}^Tq_t\left( x(t)|x(t-1),\dots x(0)\right)$. This approach is formally exact. Indeed, if we set each $q_t\left( x(t)|x(t-1),\dots x(0)\right) =\mathbb {P}\left[ x(t)|x(t-1),\dots ,x(0),\mathscr {O}\right]$, we would recover the exact posterior, due to equation (4). However, this is in practice unfeasible, because it would require $q_t$ to depend on a huge (i.e. exponential in the size of the system) number of parameters. The general idea of CVA method is to restrict the functional space of *Q* assuming the $q_t(x\left( t \right) | x \left( t-1 \right) ,\dots , x \left( 0 \right) )$ to have the same broad functional form of the unconstrained prior distribution $\mathbb {P}\left[ {x}(t)|{x}(t-1),\dots ,{x}(0)\right]$, retaining then the ability to efficiently compute it and sample from it, but generalizing it by the addition of extra parameters. This generalization will naturally allow for the spatial and/or time heterogeneity that is present in the corresponding terms in the posterior, and will be explained in detail for the specific models in the next sections. In particular, we chose in the approximating distribution of CVA to maintain the following properties (if they are present) of the prior distribution:

1. *Spatial Independence*. In agent-based models^6^ on *N* agents, $x(t)=\left( x_1(t),\dots ,x_N(t)\right)$ and most dynamical processes satisfy a spatial conditional independence property^7^, namely that:
  6 $$\begin{aligned} \mathbb {P}[x(t)|x(t-1),\dots ,x(0)] =\prod _{i=1}^{N}\mathbb {P}[x_{i}(t)|x(t-1),\ldots ,x(0)], \end{aligned}$$
  As this property is often crucial for efficient sampling from $\mathbb {P}[\textbf{x}]$, CVA maintains it on the approximating $q_t$ i.e. $q_t({x}(t)|{x}(t-1),\dots ,{x}(0))= \prod _i q^i_t(x^i(t)|{x}(t-1),\dots ,{x}(0))$.
2. *Local interactions*. Each variable of the prior process, moreover, might depend only on a restricted (local) set of variables on a given contact network; we choose to preserve this dependence in $q_t$. Note that this property is in general not present in the posterior.
3. *Markovianity*. If the prior distribution defines a memory-less stochastic process^8^, namely
  7 $$\begin{aligned} \mathbb {P}\left[ x(t)|x(t-1),\dots ,x(0)\right] =\, \mathbb {P}\left[ {x}(t)|{x}(t-1)\right] , \end{aligned}$$
  CVA extends this property to the approximating factors as well, $q^i_t\left( x^i(t)|{x}(t-1),\dots ,{x}(0)\right) =q_t^i\left( x^i(t)|{x}(t-1)\right)$. Note that if additionally the observations are time-factorized, namely $\mathbb {P}[\mathscr {O}|\textbf{x}]=\prod _{t=0}^T \mathbb {P}[\mathscr {O}_t|{x}(t)]$, then it can be shown (See Section IX of the Supplementary Information) that Markovianity extends to the posterior distribution, $\mathbb {P}[{x}({t})|{x}(t-1),\dots ,{x}(0),\mathscr {O}]=\mathbb {P}[{x}(t)|{x}({t-1}),\mathscr {O}]$.

There are simple but instructive examples where CVA leads to the exact posterior, see for example the SI epidemic model for $N=2$ individuals in Section I of the Supplementary Information.

CVA can be used to tackle some difficult problems emerging in the field of epidemic inference, such as epidemic risk assessment from partial and time-scattered observations of cases, or the detection of the sources of infection. These problems have been recently addressed within a Bayesian probabilistic framework using computational methods inspired by statistical physics^9–11^, and generative neural networks^12^. With respect to existing similar approaches based on variational autoencoders (e.g.^12,13^), the CVA ansatz for the posterior distribution does not employ neural networks, has comparatively a much smaller set of parameters and allows for much simpler physical interpretations. In particular, risk assessment from contact tracing data is of major importance for epidemic containment, because having access to an accurate measure of the individual risk can pave the way to effective targeted quarantine plans based on contact tracing devices^14–16^. Moreover, in epidemic problems, there are quantities of interest that are not known a priori. An example is the infection rate of the disease. Our method can be used to compute such quantities, treated as hyperparameters of the CVA distribution. Being able to find the prior parameters of a distribution gives also the possibility to simplify the inference problem by adopting a simpler model. For example, in the context of epidemic inference, CVA allows one to study inference problems related to the SEIR model (introduced later) with an effective SI model, which is simpler than SEIR. This part, which we call *model reduction* is illustrated in detail in the Results section. After presenting the CVA in a general setting, its main features will be discussed by exploiting a conditioned random walk^17–19^ as a toy model. Then, an application to the important problem of epidemic inference and risk assessment on dynamic contact networks is developed and analyzed in detail. We stress that the two reference cases, i.e. the epidemic inference and the conditioned random walk, represent two very different dynamical processes, the former continuous in time while the latter advances in discrete time-steps.

## Methods

The method is based on approximating the original constrained process by introducing an effective unconstrained causal process that is naturally consistent with the observations.

Let $Q_{\theta }(\textbf{x})$ be the probability distribution of a generalized dynamics, parametrized by the vector $\theta$ of parameters. The best approximation to $\mathbb {P}\left[ \textbf{x}|\mathscr {O}\right]$ (in a precise variational sense) can be obtained by observing that Eq. (1) can be interpreted as a Boltzmann distribution $Z^{-1}\exp \left[ -H\left( \textbf{x}\right) \right]$ with $H\left( \textbf{x}\right) =-\log \mathbb {P}\left[ \textbf{x},\mathscr {O}\right]$ and $Z=\mathbb {P}\left[ \mathscr {O}\right]$ and by minimizing the corresponding variational free energy^20^, i.e.

8 $$\begin{aligned}\mathscr {F}\left( Q_{\theta }\right)&:=\intop {\textrm{d}}\textbf{x}Q_{\theta }\left( \textbf{x}\right) \log \frac{Q_{\theta }\left( \textbf{x}\right) }{\mathbb {P}\left[ \textbf{x},\mathscr {O}\right] } \end{aligned}$$

9 $$\begin{aligned}&=\left\langle \log \frac{Q_{\theta }\left( \textbf{x}\right) }{\mathbb {P}\left[ \textbf{x},\mathscr {O}\right] }\right\rangle _{Q_{\theta }} . \end{aligned}$$

This quantity can be estimated efficiently by sampling the distribution $Q_{\theta }$. Note that $\mathscr {F}\left( Q_{\theta }\right) =D_{KL}\left( Q_{\theta }||\mathbb {P}\left[ \textbf{x}|\mathscr {O}\right] \right) -\log \mathbb {P}\left[ \mathscr {O}\right]$ where $D_{KL}$ is the Kullback-Leibler divergence^21^. To optimize $\mathscr {F}$, a gradient descent method can be employed (see Section V of the Supplementary Information), where the gradient can be also estimated by means of sampling, indeed

10 $$\begin{aligned} \nabla _{\theta }\mathscr {F}\left( Q_{\theta }\right)&=\left\langle \nabla _{\theta }\log Q_{\theta }\left( \textbf{x}\right) \log \frac{Q_{\theta }\left( \textbf{x}\right) }{\mathbb {P}\left[ \textbf{x},\mathscr {O}\right] }\right\rangle _{Q_{\theta }}. \end{aligned}$$

The crucial point in this estimation is that both $Q_{\theta }$ and $\mathbb {P}\left[ \textbf{x},\mathscr {O}\right] =\mathbb {P}[\textbf{x}]\mathbb {P}\left[ \mathscr {O}|\textbf{x}\right]$ have explicit expressions that, due to their causal structure, can be efficiently computed using rejection-free sampling, at difference with $\mathbb {P}\left[ \textbf{x}|\mathscr {O}\right]$ and $\mathbb {P}\left[ \mathscr {O}\right]$ which do not benefit from this property. The fact that the samples are independent allows for trivial parallelism in implementation. For a detailed description of the gradient descent optimization adopted in this work, we refer the reader to Section V of the Supplementary Information.

When a fixed point is reached, the corresponding distribution $Q_{\theta }\left( \textbf{x}\right)$ is the argument that (locally) minimizes the free energy in (9) therefore providing an approximation of the posterior distribution $\mathbb {P}\left[ \textbf{x}|\mathscr {O}\right]$. Finally, the result can be used to generate samples satisfying the constraints given by $\mathscr {O}$ and to compute interesting observables from them by efficiently computing sample averages.

### A toy model application: conditioned random walk

**Figure 1:**
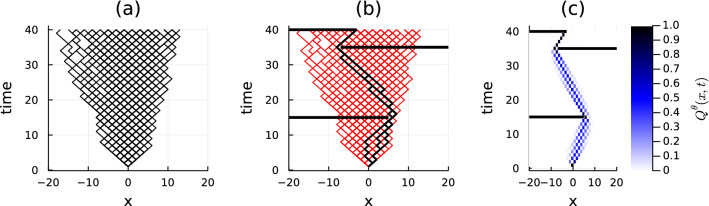
Panel (**a**) Unconditioned homogeneous random walk on a one-dimensional lattice. Time is reported on the vertical axis (up to $T=40$) and the spatial coordinate *x* is on the horizontal axis. Panel (**b**) Some trajectories are sampled from the unconditioned homogeneous distribution. The black (red) ones (do not) satisfy the constraints, i.e. they (do not) avoid the black horizontal barriers. The fraction of feasible trajectories among a given pool can be numerically estimated, and it approaches $10^{-6}$. In other words, only one of a million trajectories sampled from the unconditioned distribution satisfies the constraint. Panel (**c**) The distribution of the trajectories sampled from the CVA distribution. The color of each pixel indicates the probability for a trajectory to visit the corresponding state at a specific time.

Before introducing the main scenario where CVA is employed—i.e. on epidemic spreading models -, we first discuss a simple but instructive application of the method, that consists in generating an approximate probabilistic description for a conditioned random walk. A simple realization of the latter is a one-dimensional random walk, starting at site $x\left( 0\right) =0$. If the generating process is spatially homogeneous, the probability of every feasible trajectory $\textbf{x}$ of length *T* is $\mathbb {P}[\textbf{x}]=2^{-T}$. Note that every possible trajectory can be directly sampled by means of a causal generative process, namely a discrete-time Markov chain in which the conditional probability of a jump is $\mathbb {P}\left[ x\left( t+1\right) =x\left( t\right) \pm 1\,|x\left( t\right) \right] =1/2$. Fig. 1(a) displays a space-time representation for a set of realizations of such an unbiased random walk (black paths). For this process, let us imagine a procedure that, given a time instant $t^\mu$ and position $x^\mu$ in space, can test if the trajectory was at time $t^\mu$ to the left or right of position $x^\mu$, and denote the corresponding half-line as $W^\mu \subseteq \mathbb {Z}$. Assume that for a given unknown trajectory, we have *M* observations of this kind $\mathscr {O}=(t^\mu ,W^\mu )_\mu$ with $\mu =1,\dots ,M$. The posterior probability of a trajectory $\textbf{x}$ can be written as

11 $$\begin{aligned} \mathbb {P}\left[ \textbf{x}|\mathscr {O}\right] =\frac{\prod _{\mu =1}^M\mathbb {I}[x\left( t^\mu \right) \in W^\mu ]}{\sum _{\textbf{y}}\prod _{\mu =1}^M\mathbb {I}[y\left( t^\mu \right) \in W^\mu ]}, \end{aligned}$$

where the numerator is 1 only if the trajectory $\textbf{x}$ satisfies all observations, and zero otherwise. The denominator is the sum over all trajectories (so the variable $\textbf{y}$ runs over the space of all the possible trajectories) of the numerator and plays the role of a normalization term for the posterior. The effect of $\mathscr {O}$ is to select (or constrain to) a subset of the trajectories of a free random walk, i.e. those compatible with the observations. One could naively sample trajectories from the free dynamics and then select only those compatible with $\mathscr {O}$. However, as depicted in Fig. 1, the fraction of trajectories compatible with the constraints might be very small to allow for a feasible computation: in the example of Fig. 1(b), where it is assumed that three regions at specific time steps (black horizontal barriers) cannot be crossed, several realizations of the unconstrained dynamics are discarded (red paths), while only a small fraction is kept (black paths). In this regard, the CVA provides an efficient way of generating trajectories compatible with the constraints by building up an effective probability distribution that is - by construction - compatible with the former. Within the framework provided by CVA, the following causal *ansatz* can be introduced for the conditioned random walk problem:

12 $$\begin{aligned} Q_{\theta }\left( \textbf{x}\right) = \delta _{x\left( 0\right) ,0}\prod _{t=0}^{T-1}\left[ r_{x\left( t\right) }^{t}\delta _{x\left( t+1\right) ,x\left( t\right) +1} +l_{x\left( t\right) }^{t}\delta _{x\left( t+1\right) ,x\left( t\right) -1}\right] , \end{aligned}$$

with $\theta =\left\{ r_{x}^{t}\right\} _{x=-T,\dots , T}^{t=1,\dots , T}$ being the set of site-dependent and time-dependent rates to jump to the right, and $l_{x(t)}^{t}=1-r_{x(t)}^{t}$ the associated probabilities to jump to the left. We remark that Eq. (12) has the same functional form as the unconstrained distribution, i.e. it still represents the probability distribution of a random walk, but with heterogeneous (in general, both in space and time) jump rates. The distribution $Q_{\theta }$ requires $T\left( 2T+1\right)$ parameters, where $2T+1$ is the total number of sites that can be visited by the realizations of the random walk. These parameters are sought by minimizing the KL distance between $Q_{\theta }$ and the posterior distribution Eq. (11). The resulting probability $Q_{\theta }\left( \textbf{x} \right)$ obtained using the CVA is characterized by heterogeneous rates $r_{x}^{t}$ whose dependence in time and space perfectly mirrors the constraints introduced by the barriers. The marginal distributions of the trajectories sampled from $Q_{\theta }\left( \textbf{x}\right)$ are represented in Fig. 1(c), where the color gradient is associated with the marginal probability of occurrence of each step.

### Epidemic models and observations

From now on, we will consider a class of individual-based epidemic models describing a spreading process in a community of *N* individuals, interacting through a (possibly dynamic) contact network. The overall state of the system at time *t* (consisting of the state of each individual) is described by a vector $\textbf{x}\left( t\right) \in \mathcal {X}^{N}$, where $\mathcal {X}$ is a finite set of possible health conditions (called compartments). The simplest, but already non-trivial, model of epidemic spreading is the discrete-time Susceptible-Infected (SI) model^22^, in which $\mathcal {X}=\left\{ S,I\right\}$ (corresponding to an individual being “susceptible” and “infected”, respectively) where the only allowed transition occurs from state *S* to state *I*. More precisely, each time *t*, if an infected individual *j* is in contact with a susceptible individual *i*, the former can infect the latter (which moves into state *I*) with a transmission probability $\tilde{\lambda }_{ji}\left( t\right)$, sometimes called *transmissivity*. Since transmissions are independent, the individual transition probabilities are

13 $$\begin{aligned} \mathbb {P}\left[ x_{i}\left( t+\Delta t\right) =S|{x}\left( t\right) \right] =\delta _{x_{i}\left( t\right) ,S}\prod _{j\ne i}\left( 1-\tilde{\lambda }_{ji}\left( t\right) \delta _{x_{j}\left( t\right) ,I}\right) \end{aligned}$$

and $\mathbb {P}\left[ x_{i}\left( t+\Delta t\right) =I|{x}\left( t\right) \right] =1-\mathbb {P}\left[ x_{i}\left( t+\Delta t\right) =S|{x}\left( t\right) \right]$. A simple assumption is that time dependence only enters to describe the dynamic nature of the contact network (with $\tilde{\lambda }_{ji}\left( t\right) =0$ if there is no contact between *j* and *i* at epoch *t*). More realistically, the transmission probability $\tilde{\lambda }_{ji}\left( t\right)$ should also depend on the current stage of infection of the infector *j* (e.g. presence/absence of symptoms) and thus mainly on the time elapsed since her own infection, making the epidemic dynamics non-Markovian.

Assuming transmission probabilities proportional to $\Delta t$ in Eq. (13) and defining transmission rates as $\lambda _{ij}\left( t\right) =\lim _{\Delta t\rightarrow 0^{+}}\tilde{\lambda }_{ij}\left( t\right) /\Delta t$, a continuous-time version of the SI model is obtained. Both for the discrete-time and continuous-time SI models, the history of the epidemic process can be fully specified by the “infection times” $\left\{ t_{i}\right\} _{i=1}^{N}$ of all individuals; we conventionally set $t_{i}=0$ if individual *i* is already infected at the initial time and $t_{i}=+\infty$ if *i* is never infected during the whole epidemic process. In terms of the trajectory vector $\textbf{t}:=\left( t_{1},\dots ,t_{N}\right)$, the probability pseudo-density of an epidemic history can be generally written as

14 $$\begin{aligned} \mathbb {P}\left[ \textbf{t}\right]&= \prod _{i}\left\{ \gamma \delta \left( t_{i}\right) +\left( 1-\gamma \right) \Lambda \left( \sum _{j\ne i}\mathbb {I}\left[ t_{j}\le t\right] \lambda _{ji}\left( t\right) ,t_{i}\right) \right\} , \end{aligned}$$

where $\gamma$ denotes the probability of each individual to be a patient zero, and $\Lambda \left( f(t),b\right) =f\left( b\right) e^{-\int _{-\infty }^{b}f\left( t\right) dt}$ is the first-success distribution density of an event with rate *f*(*t*). Hence, the quantity $\Lambda \left( \sum _{j\ne i}\mathbb {I}\left[ t_{j}\le t\right] \lambda _{ji}\left( t\right) ,t_{i}\right)$ is the probability density associated with the infection, at time $t_{i}$, of individual *i* by one of its infectious contacts at previous times. Notice that when $\int _{-\infty }^{b}\Lambda \left( f,t\right) dt<1$, it means that there is a non-zero probability of the individual remaining susceptible. In that case, we will formally assign the defect mass $1-\int _{-\infty }^{b}\Lambda \left( f,t\right) dt$ to $t=\infty$. In addition to the epidemic model, a set of observations has to be defined. In real epidemics, observations mirror the outcomes of medical tests, namely the state of an individual *i* at time *t*. For the sake of simplicity, an auxiliary variable $r \in \{+. -\}$ representing positive or negative tests, respectively, is defined, such that each observation can be encoded as a triplet $\left( i,t,r\right)$. Given the stochastic nature of clinical tests, it is assumed that the outcome *r* of a test performed on individual *i* at time *t* obeys a known conditional distribution law $\mathbb {P}\left[ r|{t}_{i}\right]$ where ${t}_{i}$ represents the infection time of individual *i*. When medical tests are affected by uncertainty, i.e. there exist non-zero false positive and false negative rates of the diagnostic tests, the conditional probability states

15a $$\begin{aligned} \mathbb {P}\left[ r=+|{t}_{i}\right]&=\left( 1-p_{{\rm FNR}}\right) \,\mathbb {I}\left[ t_{i}\le t\right] +p_{{\rm FPR}}\,\mathbb {I}\left[ t_{i}>t\right] \end{aligned}$$

15b $$\begin{aligned} \mathbb {P}\left[ r=-|{t}_{i}\right]&=p_{{\rm FNR}}\,\mathbb {I}\left[ t_{i}\le t\right] +\left( 1-p_{{\rm FPR}}\right) \,\mathbb {I}\left[ t_{i} >t\right] \end{aligned}$$

For a population undergoing *M* individual test events, the set $\mathscr {O}$ of observations is then identified with the set of triplets $\left( i^{\mu },t^{\mu },r^{\mu }\right)$ for $\mu =1,\dots ,M$. As in the random walk example, each observation constrains the dynamics: in the noise-less case (i.e. $p_{{\rm FNR}}=p_{{\rm FPR}}=0$) the posterior distribution gives zero measure to all the epidemic trajectories that violate the observations. Given a realization of an epidemic model defined on a contact network, and the (possibly noisy) observation of the states of a subset of the individuals (at possibly different times), the epidemic risk assessment problem consists of estimating the epidemic risk, i.e. the risk of being infected, of the unobserved individuals at some specific time. In practice, it amounts to computing marginal probabilities from the posterior distribution

16 $$\begin{aligned} \mathbb {P}\left[ x_{i}\left( t\right) =I|\mathscr {O}\right] =\int d\textbf{t}\mathbb {I}\left[ t_{i}\le t\right] \mathbb {P}\left[ \textbf{t}|\mathscr {O}\right] \end{aligned}$$

where $\int d\textbf{t}$ denotes the integral over all infections times $t_{1},\dots ,t_{N}$.

A richer epidemic model, that is often used as a testing ground for more realistic scenarios (see e.g. Refs.^23,24^), is the SEIR model^25^, which includes also the Exposed (E) and Recovered (R) states, i.e. $\mathcal {X}=\left\{ S,E,I,R\right\}$. The only allowed transitions are $S\rightarrow E$ (representing the contagion event), $E\rightarrow I$, and $I\rightarrow R$; the latter ones occur for each individual independently of the others, with latency and recovery rates $\nu _i$ and $\mu _i$, respectively. The previous representation in terms of transmission times can be straightforwardly generalized to the SEIR model (by introducing individual-wise infective and recovery times) as well as the definition of observations from clinical tests and the measure of the individual risk (see Supplementary Information online for additional details).

The choice of the parameters $\theta$ of the CVA *ansatz* reflects and somehow generalizes the features of the generative model $\mathbb {P}[ \textbf{t}]$: in the SI case for instance, for each individual *i*, heterogeneous infection rates $\lambda _{i}\left( t\right)$, zero-patient probabilities $\gamma _{i}$, and self-infection rates $\omega _{i}(t)$ are defined. Since $\lambda _{i}\left( t\right)$ and $\omega _{i}\left( t\right)$ are time-dependent quantities, an additional parametrization is introduced for computational purposes. Then, the trial distribution $Q_{\theta }\left( \textbf{t}\right)$ is optimized with respect to the full set of parameters. The total number of parameters for the inference in the SI model in a population of *N* individuals is 7*N*, while for the SEIR model is 13*N*. We refer to Section III of the Supplementary Information for a more detailed discussion of the parameter choice and of the implementation of the gradient descent.

## Results on epidemic inference

### Performances on synthetic networks

The performance of the CVA in reconstructing epidemic trajectories can be tested by measuring its ability in classifying the state of the unobserved individuals based on their predicted risk. The results are directly compared with those obtained using other inference techniques previously proposed in the literature, such as Belief Propagation^26^ as implemented in^9^ (sib), a Monte Carlo method (MC), a Soft Margin method (soft) adapted from^27^, and simple heuristic methods based on Mean Field equations (MF)^9^ or on sampling (heu). A description of the implementation of the several methods used for comparison is provided in the Supplementary Information (Section VII) online.

**Figure 2:**
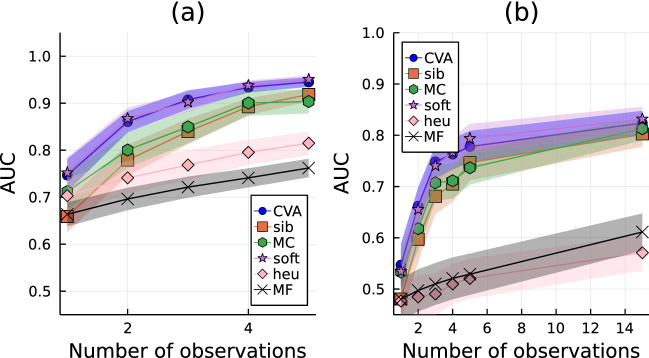
Area under the ROC (AUC) as a function of the number of observations for the risk assessment problem, i.e. $t^{\star } = T$, in panel (**a**), and for the patient-zero problem, $t^{\star } = 0$, panel (**b**). The simulated contact graph is a proximity network with average connectivity 2.2/*N*. For both simulations in panels (**a**) and (**b**), the total number of individuals is $N=50$, the probability of being the zero patient is set to $\gamma =1/N$, and the infection rate is $\lambda =0.1$. For each epidemic realization, the inference is performed for an increasing number of noiseless observations (here $p_{{\rm FNR}}=0$) at time $t_{{\rm obs}} = T$. Thick lines and shaded areas indicate the averages and the standard errors computed over 40 different instances.

For the sake of simplicity, we considered SI epidemic processes on proximity graphs^28^, i.e. random graphs generated by proximity relationships between $N = 50$ individuals randomly drawn from a uniform distribution on a two-dimensional square region (a definition of proximity graphs is given in Section VIII of the Supplementary Information). Each instance corresponds to a different realization of both the dynamical network and the forward epidemic propagation. Observations $\mathscr {O}$ are noiseless, i.e. $p_{{\rm FNR}} = 0$, and performed on a randomly chosen fraction of the population at a fixed time $t_{{\rm obs}} = T$. The comparison among the different inference methods is performed by ranking the individual marginal probabilities of being in state *I* at a chosen time $t^{*}$ and building the corresponding *receiving operating characteristic* (ROC) curve^29^. The AUC (area under the ROC curve) at a time $t^{*}$ is an indicator of the accuracy of the method in reconstructing the state of the individuals at that time. The AUC at initial time ($t^{*}=0$, patient-zero problem) and at the final time ($t^{*}=T$, risk assessment) are shown in Fig. 2 as function of the number of observations available.

As one may expect, in both cases the average performances of all methods improve when the number of observations increases. In particular, the Soft Margin method is expected to converge to the exact results for this type of experiment when the number *N* of individuals is small. The results obtained with CVA are very close (and closer than any other technique) to those obtained by means of Soft Margin (soft), even in the interesting and challenging regime with only a few observations.

To further investigate the performances of CVA against other state-of-the-art techniques, the AUC associated with the prediction of individual risk is quantified as a function of time, with two different observation protocols: (i) the states of a fraction of individuals are observed at observation times scattered over the duration of the simulation or (ii) observations are performed at the last time $t_{{\rm obs}} = T$. More precisely, in the first protocol observation times are randomly drawn a priori with uniform distribution in the interval $\left[ 1,T\right]$, and observations are biased towards tested-positive outcomes to mimic a realistic scenario where symptomatic, i.e. infected individuals, are more likely tested than susceptible ones. For these experiments, two realistic dynamic contact network instances are considered, one generated using the Spatio-temporal Epidemic Model (StEM) in continuous-time in Ref.^30^ and the other using the discrete-time OpenABM model in Ref.^14^ (see Section VIII of the Supplementary Information for a brief description of StEM and OpenABM models). For sake of simplicity, instead of adopting the complex epidemic dynamics described in Ref.^14^ and Ref.^30^, epidemic realizations are generated using a continuous-time SI model on these contact graphs.

**Figure 3:**
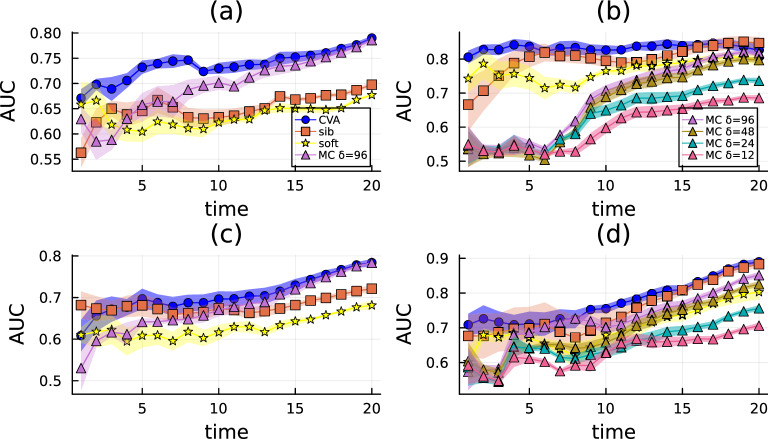
AUC associated with the prediction of the infected individuals, for the Causal Variational Approach (CVA), Belief Propagation (sib) and SoftMargin (soft), and MCMC (MC) as a function of time during the epidemic propagation of a SI model on several instances of dynamic contact network generated using the OpenABM model^14^ (in panel (**a**) $N = 2000$, in (**c**) $N = 1000$) and the StEM in Ref.^30^ (panels (**b**) and (**d**)) for $N = 904$. The infection rate is set to $\lambda =0.15$ for the latter and $\lambda = 0.02$ for the former; observations are noiseless in both cases. For panels (**c**) and (**d**), observations are performed at the last time of the dynamics, i.e. $t_{{\rm obs}} = T$. For the results in panels (**a**) and (**b**) observation times are extracted uniformly in the range $\left[ 1, T \right]$; at each observation time $t_{{\rm obs}}$, infected nodes are observed with a biased probability equal to $1.1\times N_{I}\left( t_{{\rm obs}}\right) /N$ where $N_{I}\left( t_{{\rm obs}}\right)$ is the number of infected individuals at time $t_{{\rm obs}}$ and *N* is the total number of individuals. The total number of observations is $n_{{\rm obs}} = N \cdot 0.1$ for OpenABM and $n_{{\rm obs}} = 100$ for the StEM.

A measure of the individual risk is computed according to all different methods (CVA, sib, soft, and MC), and the corresponding AUCs are shown as functions of time (in days), in Fig. 3(a, c) and (b, d) for OpenABM and the StEM respectively. For the latter only, we also consider different MC parameterizations, in particular when using $\delta \in \{12, 24, 48, 96\}$ hours; a further increase of $\delta$ does not carry any improvement of the results. The quantity $\delta$ is associated with the MC move’s proposal (see Section VII of the Supplementary Information for a detailed description). Panels (a) and (b) are associated with the observations scattered in time, while panels (c) and (d) use observations at the last time only. In panel (a) simulations are run for $N = 2000$, while in panel (c) we set $N = 1000$. It is easy to see that, in panels (a) and (c), CVA (blue dots) is the best-performing method in terms of AUC; only MC (pink triangles) reaches comparable AUC for $t \sim T$. The results achieved by Belief Propagation are similar to those produced by CVA when the size of the graph is $N = 1000$ (panel (c)), and significantly deteriorate for $N = 2000$ (panel (a)). For the instances generated according to StEM in panels (b) and (d), the comparison reveals that CVA achieves the largest values of the AUC at all times and only Belief Propagation (sib, orange squares) performs comparably to CVA for the risk assessment problem, i.e. the inference at the last time of the dynamics. MC for $\delta \in \{48, 96 \}$, approaches CVA performances in the last days while is not able to predict the zero patient. Indeed, the AUC associated with MC predictions for all parametrizations is slightly larger than 0.5 for $t < 5$ when observations are performed at the last time of the dynamics.

### Hyperparameters inference

**Figure 4:**
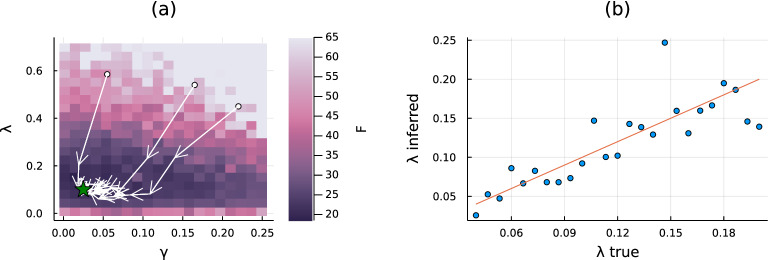
Panel (**a**) Heat map of the free energy ($F := -\log \mathbb {P}(\mathscr {O})$) computed at the convergence of CVA as a function of the assumed hyperparameters of the generative SI model. The experiment is performed on a proximity graph with $N=50$ individuals and density $\rho =2/N$; the epidemic model is characterized by the zero-patient probability $\gamma ^*=1/N$ and the infection rate $\lambda ^*=0.1$, shown here as a green star. We perform a large number of observations ($n_{{\rm obs}}=2N$) at uniformly randomly distributed times. As expected, the lowest values of this free energy are concentrated around the exact value $(\gamma ^*,\lambda ^*)$. The oriented paths (white arrows) represent the convergence towards the minimum of $-\log \mathbb {P}[\mathscr {O}]$ obtained by performing a gradient descent algorithm over the hyperparameters starting from three different initial points in the plane $(\gamma ,\lambda )$. Panel (**b**) Scatter plot of inferred values for the infection probability against the ground truth. In these experiments, we fix and assume to know the zero patient probability $\gamma =1/N$ while the infection parameter $\lambda$ is varied. For each $\lambda$ an epidemic simulation is performed and $n_{{\rm obs}}=10N$ observations are taken at uniformly randomly distributed times.

In the previous numerical experiments, the parameters of the generative SI model, i.e. the (homogeneous) infection rate $\lambda$ and the probability of being the zero patient $\gamma$, are assumed to be known. These quantities enter the CVA formalism as hyperparameters of the prior distribution which are often inaccessible in realistic applications, but can be estimated as those realizing the minimum of the free-energy $F=-\log \mathbb {P}[\mathscr {O}]$. This can be achieved by gradient descent if the number $n_{{\rm obs}}$ of available observations is sufficiently large (see Section VI of the Supplementary Information for details). An example of the quality of the parameter inference is provided by the following experiment. For a SI model with $n_{{\rm obs}}$ and true parameters $\left( \gamma ^{\star }, \lambda ^{\star }\right) = \left( 1/N, 0.1\right)$ (see the caption of Fig. 4 for further details), Fig. 4(a) shows a heatmap of *F* computed at the convergence of CVA as function of the pair of values $\left( \gamma ,\lambda \right)$ used as the hyperparameters of the corresponding prior distribution. The region attaining the lowest values also contains the true values $(\gamma ^{*},\lambda ^{*})$. The oriented paths (white arrows) in Fig. 4(a) represent the sequences of intermediate values of $\lambda$ and $\gamma$ obtained during the convergence process of CVA, starting from three different initial conditions. These traces show that trajectories end up in the same region, very close to where the true values are located (green star). Similar experiments, where the value of the zero patient probability is set to $\gamma = 1 / N$ and the infection probability varies in the range [0.05, 0.20] are performed. Similarly to the previous set-up, CVA is applied to infer the parameter $\lambda$. Figure 4(b) displays a scatter plot of the inferred values against the true ones. Results suggest a good agreement between the result of the inference and the generative process.

### Model reduction

**Figure 5:**
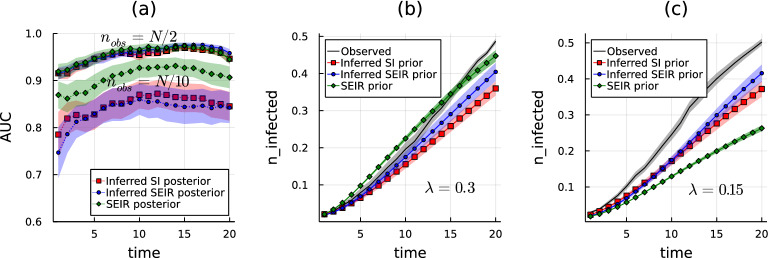
Effects of model reduction on inferential performances and generative capabilities. The numerical experiments are performed on a proximity graph with $N=100$ individuals and density 2.2/*N*. The observed epidemic realizations are generated using an SEIR model with $\gamma =1/N$, $\lambda =0.3$ (panels (**a**) and (**b**)) and 0.15 (panel (c)), latency delay $\nu =0.5$ and recovery delay $\mu =0.1$. Panel (**a**) Values of the AUC as a function of time obtained using the CVA in two observation regimes (when the number of observations is $n_{obs}=N/10$ and $n_{obs}=N/2$), with the three different inferred posterior distributions: an SEIR model with known hyperparameters (green diamonds), an SEIR model with unknown hyperparameters (blue circles), and a SI model with unknown hyperparameters (red squares). Shaded areas represent the error around the average value, computed using 22 instances. Panels (**b**) and (**c**) The average fraction of infected individuals as a function of time estimated using the correct SEIR prior model (green diamonds), an SEIR prior with the inferred hyperparameters (blue circles), and a SI prior model with the inferred hyperparameters (red squares). The regimes shown correspond to unbiased observations (center, for $\lambda =0.3$), and to observations preferentially sampled from large outbreaks (right, for $\lambda =0.15$). The black curves represent the same quantity computed from the observed epidemic realizations. Shaded areas represent the standard error computed from 40 realizations of the dynamics.

For viral diseases with sufficiently known transmission mechanisms, agent-based modeling using discrete-state stochastic processes has proven useful to build large-scale simulators of epidemic outbreaks and design containment strategies^24,30^. Such mathematical models are much more complex than the SI model analyzed previously, as they need to include additional specific features of real-world diseases. In particular, models may assume different infected states, characterized by a different capability of transmitting the virus and diverse sensitivity to diagnostic tests. Another important feature that can emerge from realistic transmissions is that individuals can stop being infectious, even before recovering from the infected state, because of the decay of their viral load. These ingredients may be effectively included in the SI and SEIR models by introducing time-dependent infection rates, which is a natural assumption in the framework of the Causal Variational Approach (see Section II-III of the Supplementary Information). This property makes the latter a very suitable inference method to approximate unknown and possibly complex generative epidemic processes using classes of simpler probabilistic models. A simple test of such a potentiality is provided by the following example. Several epidemic realizations are generated with an SEIR prior model and the quality of the inference obtained by the Causal Variational Approach is evaluated when (i) the SEIR model is also used as an *ansatz* for the posterior distribution and (ii) when the posterior distribution is approximated with a simpler probabilistic model, such as the SI model. If the parameters of the generative SEIR model are known, the hyperparameters of the SEIR posterior are also known. The corresponding results (green diamonds) for the AUC as a function of time on a proximity graph are displayed in Fig. 5(a). Otherwise, the hyperparameters of the SEIR posterior can be inferred by means of the CVA (blue circles). Finally, the *ansatz* for the posterior distribution can be simplified to a SI model, and the corresponding hyperparameters can be inferred as well within the CVA (red squares). The overall quality of the inference depends on the possibly different regimes of information contained in the observations. Strikingly, when the generative model is not known, the results from SEIR-based and SI-based inference are always very close to each other. For a sufficiently large number of observations, such results are also close to those obtained with the SEIR posterior and known hyperparameters.

From a generative perspective, the inferred hyperparameters can be also interpreted as the epidemic parameters of some prior model, from which epidemic realizations can be sampled. It is natural to ask what are the statistical properties of such generative processes compared to the original one, from which the observations were sampled. Figure 5 also shows, in two different regimes, as a function of time the average number of infected individuals estimated from the original SEIR prior model (green diamond), the SEIR prior model with inferred hyperparameters (blue circles) and the SI prior model with inferred hyperparameters (red squares). The average number of infected individuals computed over the realizations from which the observations are sampled is also displayed (black line). The regimes shown in Fig. 5 correspond to unbiased observations (panel (b), for $\lambda =0.3$), and to observations preferentially sampled from large outbreaks (panel (c), for $\lambda =0.15$). Although the discrepancy between the different curves is significant, the moderate difference between predictions obtained using SEIR and SI prior models with inferred hyperparameters suggests that model reduction is only a minor source of information bias.

## Conclusions

Sampling from the posterior distribution of a conditioned dynamical process can be computationally hard. In this work, a novel computational method to accomplish this task, called the Causal Variational Approach, was put forward. The Causal Variational Approach is based on the idea of inferring the posterior with an effective, unconditioned, dynamical model, whose parameters can be learned by minimizing a corresponding free energy functional. An insight into the potential of the method is obtained by analyzing a one-dimensional conditioned random walk in which some regions of space are forbidden. The CVA produces a generalized random walk process, with space-dependent and time-dependent jump rates, whose unconditioned realizations satisfy the imposed constraints. An application of greater practical interest concerns epidemic inference, in particular the risk assessment from partial and time-scattered observations. For simple stochastic epidemic models, such as SI and SEIR, taking place on contact networks of moderately small size, the CVA performs better or as well as the best methods currently available. Moreover, the variational nature of the method allows one to estimate the parameters of the original epidemic model that generated the observations, which enter the CVA in the form of hyperparameters. Since the CVA approximates the posterior distribution of the epidemic process by learning a set of generalized individual-based, time-dependent parameters, even with a rather simple *ansatz* for the epidemic model, such as an SI model, inference from observations coming from more complex epidemic processes can be performed. In fact, a generalized SI model with time-dependent infection rates and self-infection rates allows one to accommodate many features of real-world epidemic diseases, such as time-varying viral load and transmissivity, incubation, and recovery. The performances of the Causal Variational Approach do not seem to suffer from model reduction from SEIR to SI, suggesting that simplified epidemic models could be effective for inference also in real-world cases. The Causal Variational Approach is very flexible and, employing sampling to perform estimates, it can be applied virtually to any dynamics for which the latter can be carried out efficiently. In particular, the method can thus be applied to inference problems involving recurrent epidemic processes, such as the SIS model^31^ or other models (e.g.^32^) where immunity decays over time. There are, however, some limitations. The CVA relies on the fact that the functional form of the posterior should be similar to the one of the prior. This is not true in general. For example, let us take an epidemic SI model in which the zero-patient probability $\gamma$ is infinitesimally small. If one individual is tested positive at a certain time, then the posterior distribution is substantially different from the prior. In particular, in the prior process each individual is the patient zero independently with probability $\gamma$, while in the posterior there is a strong (anti)correlation: indeed the measure will concentrate on trajectories with exactly one infected individual (and this is impossible to reproduce with independent patient zero probabilities). Of course, this example is extremely contrived, as the probability that infection occurs at all in this system, and thus such a test result can be obtained, is infinitesimally small as well. Moreover, in this case the problem can be simply solved by adopting a more natural distribution for the initial state (either by using a non-infinitesimal initial infection probability in the prior, or by adopting a single initial infection in the test distribution *q*, see also Supplementary Information). Nevertheless, it is a simple example in which the prior functional form is substantially different from the one of the posterior.

## Supplementary Information

Supplementary Information.

## Data Availability

All data is generated using simulations and can be reproduced by following the prescriptions provided in the main text and in Supplementary Information online. A public GitHub repository containing a Julia implementation of the algorithm and notebooks to reproduce the results of this work is available at https://github.com/abraunst/Causality.git.

## References

[1] Newman, M. E. J. & Barkema, G. T. *Monte Carlo Methods in Statistical Physics* (Clarendon Press, Oxford, 1999).

[2] MacKay, D. J. *Information Theory, Inference and Learning Algorithms* (Cambridge University Press, Cambridge, 2003).

[3] Biroli, G. & Kurchan, J. Metastable states in glassy systems. *Phys. Rev. E* **64**, 016101. 10.1103/PhysRevE.64.016101 (2001). 10.1103/PhysRevE.64.016101 11461325

[4] James, R. G., Ayala, B. D. M., Zakirov, B. & Crutchfield, J. P. *Modes of information flow* 1808.06723 (2018).

[5] Sattari, S. *et al.* Modes of information flow in collective cohesion. *Sci. Adv.* **8**, eabj1720. 10.1126/sciadv.abj1720 (2022). 35138896 10.1126/sciadv.abj1720 PMC8827646

[6] Macal, C. M. & North, M. J. Agent-based modeling and simulation. In *Proceedings of the 2009 Winter Simulation Conference (WSC)*, 86–98. 10.1109/WSC.2009.5429318 (2009).

[7] Dawid, A. P. Conditional independence in statistical theory. *J. R. Stat. Soc. Ser. B (Methodological)* **41**, 1–15. 10.1111/j.2517-6161.1979.tb01052.x (1979).

[8] Norris, J. R. *Markov Chains* (Cambridge University Press, Cambridge, 1998).

[9] Baker, A. *et al.* Epidemic mitigation by statistical inference from contact tracing data. *Proc. Natl. Acad. Sci.* **118**, e2106548118. 10.1073/pnas.2106548118 (2021). 34312253 10.1073/pnas.2106548118 PMC8364197

[10] Herbrich, R., Rastogi, R. & Vollgraf, R. CRISP: A Probabilistic Model for Individual-Level COVID-19 Infection Risk Estimation Based on Contact Data, 10.48550/arXiv.2006.04942 (2022). arXiv:2006.04942 [cs, stat].

[11] O’Neill, P. D. A tutorial introduction to Bayesian inference for stochastic epidemic models using Markov chain Monte Carlo methods. *Math. Biosci.* **180**, 103–114. 10.1016/S0025-5564(02)00109-8 (2002). 12387918 10.1016/s0025-5564(02)00109-8

[12] Biazzo, I., Braunstein, A., Dall’Asta, L. & Mazza, F. A Bayesian generative neural network framework for epidemic inference problems. *Sci. Rep.* **12**, 19673. 10.1038/s41598-022-20898-x (2022). 36385141 10.1038/s41598-022-20898-x PMC9667449

[13] Wu, D., Wang, L. & Zhang, P. Solving statistical mechanics using variational autoregressive networks. *Phys. Rev. Lett.* **122**, 080602. 10.1103/PhysRevLett.122.080602 (2019). 30932595 10.1103/PhysRevLett.122.080602

[14] Ferretti, L. *et al.* Quantifying SARS-CoV-2 transmission suggests epidemic control with digital contact tracing. *Science* **368**, eabb6936. 10.1126/science.abb6936 (2020). 32234805 10.1126/science.abb6936 PMC7164555

[15] Cencetti, G. *et al.* Digital proximity tracing on empirical contact networks for pandemic control. *Nat. Commun.* **12**, 1655. 10.1038/s41467-021-21809-w (2021). 33712583 10.1038/s41467-021-21809-w PMC7955065

[16] Eames, K. T. D. & Keeling, M. J. Contact tracing and disease control. *Proc. R. Soc. Lond. Ser. B Biol. Sci.* **270**, 2565–2571. 10.1098/rspb.2003.2554 (2003). 10.1098/rspb.2003.2554 PMC1691540 14728778

[17] Foss, S. & Sakhanenko, A. Structural properties of conditioned random walks on integer lattices with random local constraints. In *In and Out of Equilibrium 3: Celebrating Vladas Sidoravicius. Progress in Probability* (eds Vares, M. E. *et al.*) 407–438 (Springer, Cham, 2021). 10.1007/978-3-030-60754-8_19.

[18] Gantert, N., Popov, S. & Vachkovskaia, M. On the range of a two-dimensional conditioned simple random walk. *Ann. Henri Lebesgue* **2**, 349–368. 10.5802/ahl.20 (2019).

[19] Ding, J., Fukushima, R., Sun, R. & Xu, C. Geometry of the random walk range conditioned on survival among Bernoulli obstacles. *Probab. Theory Relat. Fields* **177**, 91–145. 10.1007/s00440-019-00943-z (2020).

[20] Parisi, G. *Statistical Field Theory* (Avalon Publishing, London, 1998).

[21] Joyce, J. M. Kullback–Leibler Divergence. In *International Encyclopedia of Statistical Science* (ed. Lovric, M.) 720–722 (Springer, Berlin, 2011). 10.1007/978-3-642-04898-2_327.

[22] Allen, L. J. S. Some discrete-time SI, SIR, and SIS epidemic models. *Math. Biosci.* **124**, 83–105. 10.1016/0025-5564(94)90025-6 (1994). 7827425 10.1016/0025-5564(94)90025-6

[23] Kerr, C. C. *et al.* Covasim: An agent-based model of Covid-19 dynamics and interventions. *PLOS Comput. Biol.* **17**, e1009149 (2021). 34310589 10.1371/journal.pcbi.1009149 PMC8341708

[24] Hinch, R. *et al.* OpenABM-covid19-an agent-based model for non-pharmaceutical interventions against COVID-19 including contact tracing. *PLOS Comput. Biol.* **17**, e1009146. 10.1371/journal.pcbi.1009146 (2021). 34252083 10.1371/journal.pcbi.1009146 PMC8328312

[25] Biswas, M. H. A., Paiva, L. T. & Pinho, M. D. A SEIR model for control of infectious diseases with constraints. *Math. Biosci. Eng.* **11**, 761–784. 10.3934/mbe.2014.11.761 (2014).

[26] Mézard, M. & Montanari, A. *Information, Physics, and Computation* (Oxford University Press, Oxford, 2009).

[27] Antulov-Fantulin, N., Lancčić, A., Šmuc, T., Štefančić, H. & Šikić, M. Identification of patient zero in static and temporal networks: Robustness and limitations. Phys. Rev. Lett. **114**, 248701. 10.1103/PhysRevLett.114.248701 (2015). 10.1103/PhysRevLett.114.248701 26197016

[28] Mathieson, L. & Moscato, P. An introduction to proximity graphs. In *Business and Consumer Analytics: New Ideas* (eds Moscato, P. & de Vries, N. J.) 213–233 (Springer, Cham, 2019). 10.1007/978-3-030-06222-4_4.

[29] Fawcett, T. An introduction to ROC analysis. *Pattern Recognit. Lett.* **27**, 861–874. 10.1016/j.patrec.2005.10.010 (2006).

[30] Lorch, L. *et al.* Quantifying the effects of contact tracing, testing, and containment measures in the presence of infection hotspots. *ACM Transactions on Spatial Algorithms and Systems*10.1145/3530774 (2022).

[31] Ortega, E., Machado, D. & Lage-Castellanos, A. Dynamics of epidemics from cavity master equations: Susceptible-infectious-susceptible models. *Phys. Rev. E* **105**, 024308. 10.1103/PhysRevE.105.024308 (2022). 35291082 10.1103/PhysRevE.105.024308

[32] Wonham, M. J., de Camino-Beck, T. & Lewis, M. A. An epidemiological model for West Nile virus: invasion analysis and control applications. *Proc. R. Soc. Lond. Ser. B Biol. Sci.* **271**, 501–507. 10.1098/rspb.2003.2608 (2004). 10.1098/rspb.2003.2608 PMC1691622 15129960

