# Correction: Inference in conditioned dynamics through causality restoration

**DOI:** 10.1038/s41598-026-57627-7

**Published:** 2026-06-19

**Authors:** Alfredo Braunstein, Giovanni Catania, Luca Dall’Asta, Matteo Mariani, Anna Paola Muntoni

**Affiliations:** 1 DISAT, Politecnico di Torino, Corso Duca Degli Abruzzi 24, 10129 Turin, Italy; 2 INFN, Sezione di Torino, Turin, Italy; 3 Italian Institute for Genomic Medicine, IRCCS Candiolo, SP‑142, 10060 Candiolo, TO Italy; 4 Departamento de Física Téorica I, Universidad Complutense, 28040 Madrid, Spain; 5 Collegio Carlo Alberto, P.za Arbarello 8, 10122 Turin, Italy

Correction to: *Scientific Reports* 10.1038/s41598-023-33770-3, published online 05 May 2023

The original version of this Article contains an error in the Supplementary Information file, where the analytic derivation presented in Section VI of the Supplementary Information was incorrect.

The correct Supplementary Information file is now linked to this correction notice.

## Supplementary Information

Supplementary Information.

