# Supplementary material for: Correction: Inference in conditioned dynamics through causality restoration
Source: Sci Rep. 2026 Jun 19;16:19170. doi: 10.1038/s41598-026-57627-7 (PMC13282370; doi:10.1038/s41598-026-57627-7)
Supplement: Supplementary file 1 — Supplementary Information. [file 41598_2026_57627_MOESM1_ESM.pdf]

Supplementary Information for: Inference in conditioned dynamics through causality restoration

Alfredo Braunstein,[1,2,3] Giovanni Catania,[4] Luca Dall'Asta,[1,2,3,5] Matteo Mariani,[1,*] and Anna Paola Muntoni[1,3]

[1] *DISAT, Politecnico di Torino, Corso Duca Degli Abruzzi 24, 10129 Torino*

[2] *INFN, Sezione di Torino, Torino, Italy*

[3] *Italian Institute for Genomic Medicine, IRCCS Candiolo, SP-142, I-10060 Candiolo (TO), Italy*

[4] *Departamento de Física Téorica I, Universidad Complutense, 28040 Madrid, Spain*

[5] *Collegio Carlo Alberto, P.za Arbarello 8, 10122, Torino, Italy*

S I. EXACT POSTERIOR DISTRIBUTION OF A SI MODEL WITH TWO INDIVIDUALS AND ONE OBSERVATION

A simple but instructive example to understand how the Causal Variational Approach can obtain good approximations of the posterior distribution in epidemic processes is provided by analyzing the case of a continuous-time SI model with only two individuals, A and B. In this example, we assume that (i) both individuals have the same probability γ of being initially infected and (ii) they can infect each other, if infectious, with the same constant infection rate λ. A graphical representation of the role played by these parameters is shown in Figure S1 (*left*).

Suppose an individual A is observed at time $t_{\text{obs}} = T$ in the infected state (e.g., performing a clinical test), then there are two possible explanations for such an observation: either A was already infected at time $t = 0$ or A got infected by B at a time $t < T$. In both cases, the observation forces the dynamics to satisfy the constraint that A is in state I at time T. One can speculate that the observation breaks the causality property of the dynamics because a constraint set on the future (time T) affects the dynamics at previous times. If, in fact, A is not the zero patient, then B must have infected A at previous times. The effective posterior infection rate from B to A, therefore, must diverge when $t \to T$.

The epidemic trajectory can be described defining $\boldsymbol{t} := (t_{\text{A}}, t_{\text{B}})$, where t_{A} and t_{B} are the infection times of the

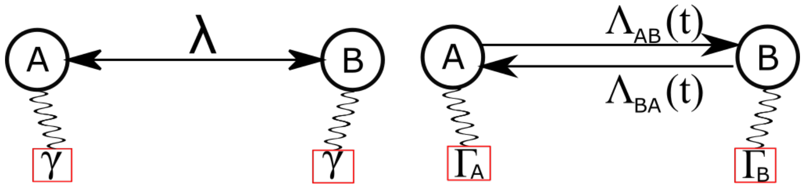

FIG. S1. Schematic representation of the role played by the epidemic parameters in a SI model with two individuals. *Left*: In the original SI model, individuals A and B have the same probability γ of being initially infected, and the same infection rate λ. *Right*: in the prior SI model used to compute the posterior by means of the CVA, γ are replaced by possibly different probabilities Γ_A, Γ_B and the infection rate λ is replaced by time-dependent rates $\Lambda_{\text{AB}}(t), \Lambda_{\text{BA}}(t)$. These parameters are inferred by minimizing the KL divergence in Eq. (8) in the main text.

* matteo.mariani@polito.it

two individuals. In terms of $\boldsymbol{t}$, the SI prior distribution can be written as

$$\mathbb{P}[\boldsymbol{t}] = \begin{cases} \gamma^2 & t_\text{A} = t_\text{B} = 0, \\ (1-\gamma)^2 & t_\text{A} = t_\text{B} = \infty, \\ \gamma(1-\gamma)e^{-\lambda t}\lambda & t_\text{A} = t \text{ and } t_\text{B} = 0\,; t_\text{A} = 0 \text{ and } t_\text{B} = t\ , \\ 0 & \text{otherwise.} \end{cases} \tag{1}$$

This prior probability distribution [1] is normalized, indeed

$$\gamma^2 + 2\gamma(1-\gamma)\lambda \int_0^\infty e^{-\lambda t} dt + (1-\gamma)^2 = \gamma^2 + 2\gamma(1-\gamma) + (1-\gamma)^2 = 1. \tag{2}$$

The observation of the state of individual A implies that

$$\mathbb{P}[\mathcal{O}|\boldsymbol{t}] = \begin{cases} 0 & \text{if } t_\text{A} > T \\ 1 & \text{otherwise.} \end{cases} \tag{3}$$

The posterior distribution $\mathbb{P}[\boldsymbol{t}|\mathcal{O}]$ can be computed using Bayes theorem as

$$\mathbb{P}[\boldsymbol{t}|\mathcal{O}] = \frac{\mathbb{P}[\boldsymbol{t}]\mathbb{P}[\mathcal{O}|\boldsymbol{t}]}{\mathbb{P}[\mathcal{O}]} \tag{4}$$

$$= \frac{1}{\mathbb{P}[\mathcal{O}]} \begin{cases} \gamma^2 & t_\text{A} = t_\text{B} = 0 \\ \gamma(1-\gamma)e^{-\lambda t_\text{A}}\lambda & 0 < t_\text{A} < T \text{ and } t_\text{B} = 0 \\ \gamma(1-\gamma)e^{-\lambda t_\text{B}}\lambda & t_\text{A} = 0 \\ 0 & \text{otherwise.} \end{cases} \tag{5}$$

where the denominator is given by

$$\begin{aligned} \mathbb{P}[\mathcal{O}] &= \gamma^2 + \int_0^T dt_\text{A}\, p_\text{AB}(t_\text{A}, 0) + \int_0^\infty dt_\text{B}\, p_\text{AB}(0, t_\text{B}) \\ &= \gamma^2 + \gamma(1-\gamma)\left(2 - e^{-\lambda T}\right). \end{aligned} \tag{6}$$

It is convenient to compute the posterior probability that individuals are infected at the initial time given the observation $\mathcal{O}$,

$$\mathbb{P}[t_\text{A} = 0|\mathcal{O}] = \frac{\gamma}{\gamma^2 + \gamma(1-\gamma)\left(2 - e^{-\lambda T}\right)} =: \Gamma_\text{A} \tag{7}$$

$$\mathbb{P}[t_\text{B} = 0|\mathcal{O}] = \frac{\gamma + (1-\gamma)\left(1 - e^{-\lambda T}\right)}{\gamma + (1-\gamma)\left(2 - e^{-\lambda T}\right)} =: \Gamma_\text{B} \tag{8}$$

For both individuals, non-causal effects arise as the infection probabilities at time 0 also depend on the infection rate λ and on the observation time T. Moreover, the expressions of $\Gamma_\text{A}, \Gamma_\text{B}$ are different because the observation on A has broken the symmetry between the two individuals.

---

[1] There is a little abuse of notation. $p_\text{AB}(0,0)$ and $p_\text{AB}(\infty,\infty)$ are probabilities, while the other term is a density of probability. The point is that the event of having two zero patients or no infections have finite probability, while the event of having a particular infection time is infinitesimal in probability and must be integrated over time.

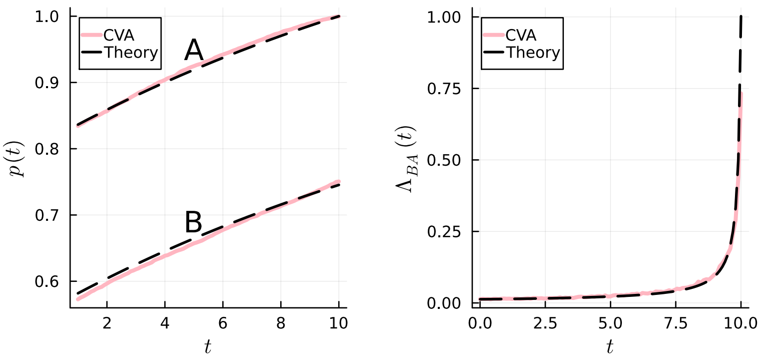

FIG. S2. . Comparison between the exact calculations (dashed black lines) and results obtained using the CVA (red lines) for a SI model ($\lambda = 0.05$ and $\gamma = 1/2$) with two individuals A and B, where A is observed infected at the final time $T = 10$. *Left*: Marginal probabilities of being infected for individuals A and B as a function of time. Due to the observation on A at time T, the two marginals are different: in particular, individual A's marginal reaches 1 at time T in such a way as to be consistent with the observation. *Right*: Effective infection rate $\Lambda_{\mathrm{BA}}(t)$ as function of time. The divergence is due to the observation on individual A at time T.

It is also possible to compute the posterior probability that individual B is infected at time t, which implies that A is initially infected, namely

$$\begin{aligned}\Lambda_{\mathrm{AB}}(t) &:= \lim_{\epsilon\to 0}\frac{\mathbb{P}[t_{\mathrm{B}} = t+\epsilon, t_{\mathrm{A}} = 0|\mathcal{O}]}{\mathbb{P}[t_{\mathrm{B}} > t, t_{\mathrm{A}} = 0|\mathcal{O}]}\\ &= \lim_{\epsilon\to 0}\frac{\gamma(1-\gamma)e^{-\lambda(t+\epsilon)}\lambda}{\gamma(1-\gamma)e^{-\lambda t}} = \lambda \end{aligned}\tag{9}$$

and the posterior probability that individual A is infected at time t by B, i.e.

$$\begin{aligned}\Lambda_{\mathrm{BA}}(t) &:= \lim_{\epsilon\to 0}\frac{\mathbb{P}[t_{\mathrm{A}} = t+\epsilon, t_{\mathrm{B}} = 0|\mathcal{O}]}{\mathbb{P}[t_{\mathrm{A}} > t, t_{\mathrm{B}} = 0|\mathcal{O}]}\\ &= \lim_{\epsilon\to 0}\frac{\gamma(1-\gamma)e^{-\lambda(t+\epsilon)}\lambda}{\gamma(1-\gamma)\lambda\int_t^T e^{-\lambda s}ds} =\\ &= \lim_{\epsilon\to 0}\frac{e^{-\lambda(t+\epsilon)}\lambda}{(e^{-\lambda t}-e^{-\lambda T})} = \frac{\lambda e^{-\lambda t}}{e^{-\lambda t}-e^{-\lambda T}}. \end{aligned}\tag{10}$$

The two quantities differ because here the observation forces individual B to infect A before the observation time $t_{\mathrm{obs}} = T$, causing the infection rate $\Lambda_{\mathrm{BA}}(t)$ to diverge when $t \to T$.

The exact posterior distribution (5) can be expressed in the form of a generalized SI model with asymmetric probabilities Γ_{A} and Γ_{B} that the individuals are already infected at the initial time and time-dependent infection rates $\Lambda_{\mathrm{AB}}(t)$ and $\Lambda_{\mathrm{BA}}(t)$. A schematic representation of the role played by these parameters as compared with the ones of the original SI model is provided in Fig. S1 (*right*). Such a generalized SI model can be used as an ansatz distribution Q_θ in the CVA. The minimum of the corresponding free energy is obtained when the generalized parameters $\Gamma_{\mathrm{A}}, \Gamma_{\mathrm{B}}, \Lambda_{\mathrm{AB}}(t)$ and $\Lambda_{\mathrm{BA}}(t)$ assume exactly the form derived above in Eqs. (7),(8),(9) and (10). It follows that the CVA provides a formally exact solution to the inference problem under study. This is shown in Figure S2, where the exact solution of the model is compared with the solution found using the CVA.

S II. PROBABILISTIC DESCRIPTION OF THE SEIR MODEL WITH OBSERVATIONS

The Susceptible-Exposed-Infected-Recovered (SEIR) model is a generalization of the SI model in which incubation (E) and recovery (R) are included. The only allowed transitions are $S \to E$, $E \to I$, $I \to R$. A transmission event by an infected individual j brings a susceptible individual i into the E state with a rate λ_{ji}, then the transition to I occurs independently of the rest of the system with rate ν_i. Finally, an infected individual i recovers, again independently of the others, with rate μ_i. In this model, the trajectory of the epidemic process is fully specified by three times: $t_i^E \leq t_i^I \leq t_i^R \in \mathbb{R}_{\geq 0}$, representing the times in which individual i enters the states E, I and R, respectively. For an initially infected individual $t_i^E = t_i^I = 0$. In this notation, the probability weight of the trajectory $\boldsymbol{t} = \left\{(t_i^E, t_i^I, t_i^R)\right\}_{i=1}^N$ can be written as

$$\mathbb{P}[\boldsymbol{t}] = \prod_i \left[\gamma\delta\left(t_i^E\right)\delta\left(t_i^I\right) + (1-\gamma)\Lambda\left(\sum_{j\neq i}\left[t_j^I \leq t \leq t_j^R\right]\lambda_{ji}(t), t_i^E\right)\Lambda\left(\nu_i\mathbb{I}\left[t_i^E \leq t\right], t_i^I\right)\right]\Lambda\left(\mu_i\mathbb{I}\left[t_i^I \leq t\right], t_i^R\right) \quad (11)$$

Test-based observations can be defined by generalizing the argument discussed in Section 3 in the main text for the SI model. Once again, the simplest realization of test r consists of a noisy evaluation of the individual's infection state, whose outcome obeys the following stochastic expression, conditioned w.r.t. the time trajectory of individual i:

$$\begin{aligned}\mathbb{P}[r=+|t_i] &= (1-p_{\mathrm{FNR}})\,\mathbb{I}\left[t_i^E \leq t < t_i^R\right] + p_{\mathrm{FPR}}\left(\mathbb{I}\left[t < t_i^E\right] + \mathbb{I}\left[t \geq t_i^R\right]\right)\\ \mathbb{P}[r=-|t_i] &= p_{\mathrm{FNR}}\,\mathbb{I}\left[t_i^E \leq t < t_i^R\right] + (1-p_{\mathrm{FPR}})\left(\mathbb{I}\left[t < t_i^E\right] + \mathbb{I}\left[t \geq t_i^R\right]\right)\end{aligned} \quad (12a)$$

where p_{FPR} (resp. p_{FNR}) is the false positive (resp. negative) ratio of the test. Here it was implicitly assumed that the test outcome does not depend on any acquired immunity.

Finally, the definition of the risk measure in Eq. (16) in the main text can be straightforwardly generalized to the SEIR model

$$\mathbb{P}[x_i(t) = I|\mathcal{O}] = \int d\boldsymbol{t}\,\mathbb{I}\left[t_i^I \leq t < t_i^R\right]\mathbb{P}[\boldsymbol{t}|\mathcal{O}] \quad (13)$$

where $\int d\boldsymbol{t}$ denotes the integral over all transition time triplets $(t_1^E, t_1^I, t_1^R), \ldots, (t_N^E, t_N^I, t_N^R)$.

S III. PARAMETRIZATION IN THE CAUSAL VARIATIONAL APPROACH

A strong advantage of CVA is its flexibility in the parametrization of the solution ansatz. It is sufficient, indeed, to weakly generalize the prior model in order to obtain the form of the $Q_\theta(\boldsymbol{x})$ to optimize. This section describes the parametrizations used for the three models whose results are presented in the main text, namely the Random Walk and the epidemic SI and SEIR models.

Random Walk – The Random Walk generative model is a discrete-time stochastic process, where at each time the walker chooses to jump right or left with a uniform probability 1/2. As discussed in Section 2 of the main text, the goal is to characterize the probability distribution of a constrained random walk. The CVA simply consists in assuming a more comprehensive parametrization of the generative model, in a way that, the conditioned distribution thus obtained still describes a Random Walk. In particular, instead of fixing the jump probability to 1/2, it is allowed to take different, heterogeneous values, depending on time and space. The parameters of the $Q_\theta(\boldsymbol{x})$ are then simply identified with probabilities r_i^t to jump to the right for a walker that at time t is in position $x(t) = i$. The ansatz has therefore the following form,

$$Q_\theta(\boldsymbol{x}) = \prod_{t=0}^{T-1}\left[r_{x(t)}^t\delta_{x(t+1),x(t)+1} + \left(1 - r_{x(t)}^t\right)\delta_{x(t+1),x(t)-1}\right] \quad (14)$$

which describes an inhomogeneous time-dependent random walk. CVA reduces to optimize Q_θ over the probabilities $\{r_i^t\}_{i=1,\dots,N}^{t=1,\dots,T}$.

Homogeneous Markovian SI model – To characterize a homogeneous and Markovian SI dynamic one needs to specify the constant infection rate λ, together with the probability γ of being the sources of the infection at the initial time. Thus, the probability of the infection times in Eq. (14) of the main text reads

$$\mathbb{P}\left[\boldsymbol{t}\right] = \prod_i \left\{ \gamma\delta\left(t_i\right) + \left(1-\gamma\right)\Lambda\left(\sum_{j\neq i}\mathbb{I}\left[t_j\leq t\right]\lambda, t_i\right)\right\}. \tag{15}$$

The Causality ansatz for this model is obtained by introducing, for each individual i, effective infection rates $\{\lambda_i(t)\}_{i=1,\dots,N}$ and zero-patient probabilities $\{\gamma_i\}_{i=1,\dots,N}$ respectively. In other words, the conditioned, and constrained, SI dynamical model is approximated with an unconditioned but inhomogeneous and time-dependent SI model. Each $\lambda_i(t)$ refers to the 'incoming' infection rate at site i at time t, namely, we impose that $\lambda_{ji}\left(t\right) = \lambda_i\left(t\right)$ for every $j \in \partial i$. The benefit carried by this parametrization is twofold: (i) this individual infection allows us to simplify the calculations and (ii) it guarantees 'outgoing' heterogeneous infection rates. A simple example can be used to illustrate this parametrization. Suppose to consider an individual i that has only two contacts with j_1 and j_2, observed to be respectively I and S at time τ. Since j_2 is susceptible at time τ, none of its previous contacts has infected it at previous times; in our parametrization, this case can be encoded by setting $\lambda_{j_2}\left(t\right) = 0$ for $t < \tau$. To cope with the observation of the state of j_1 one can tune $\lambda_{j_1}(t)$ (for $t < \tau$) to sufficiently high values to guarantee that j_1 is in state I at the observation time. In the present work, self-infection rates $\{\omega_i\}_{i=1,\dots,N}$ are also introduced, that is the rates with which individuals can get infected without any contact with an infectious individual. Although this transition is not contemplated in the generative model, it is conveniently included to satisfy some constraints associated with the observation of infected individuals, that are hard to justify through the infection rates alone. In this case, the CVA ansatz reads

$$Q_\theta(\boldsymbol{x}) = \prod_i \left\{ \gamma_i\delta\left(t_i\right) + \left(1-\gamma_i\right)\Lambda\left(\sum_{j\in\partial i}\mathbb{I}\left[t_j^I\leq t\right]\lambda_i\left(t\right) + \omega_i\left(t\right), t_i\right)\right\}, \tag{16}$$

Notice that Eq. (16) is almost identical to (14) of the main text, except for the presence of the self-infection rates. The SI model considered here is continuous in time, so that the variational parameters λ_i and ω_i should in principle be treated as continuous functions over time as well.

Since it is not possible to optimize over a function defined on a finite real domain, it is more convenient to express such rates using a family of functions, defined by a set of parameters, and optimize over the latter. A simple choice - adopted in all the results presented in the main text - is a Gaussian-like (not normalized) function for each site-dependent rate λ_i and ω_i. Each Gaussian rate will thus depend on three parameters, namely the value of the peak (labeled using μ), the standard deviation (σ), and a scale factor (p). For instance, the infection rate $\lambda_i(t)$ reads

$$\lambda_i\left(t\right) = \lambda_i^p \exp\left[-\left(\frac{t-\lambda_i^\mu}{\lambda_i^\sigma}\right)^2\right], \tag{17}$$

and analogous expressions hold for the self-infection rates $\omega_i(t)$ with parameters $(\omega_i^p, \omega_i^\mu, \omega_i^\sigma)$. With this choice, the total set of parameters to optimize over is given by:

$$\theta = \{\lambda_i^p, \lambda_i^\mu, \lambda_i^\sigma, \omega_i^p, \omega_i^\mu, \omega_i^\sigma, \gamma_i\}_{i=1,\dots,N} \tag{18}$$

Homogeneous Non-Markovian SI model – A more realistic scenario is that depicted by the homogeneous Non-Markovian SI model, where the infection rates are not individual-dependent (e.g. spatially homogeneous) but they can vary in time. In this case, Eq. (14) of the main text slightly modifies as

$$\mathbb{P}\left[\boldsymbol{t}\right] = \prod_i \left\{ \gamma\delta\left(t_i\right) + \left(1-\gamma\right)\Lambda\left(\sum_{j\neq i}\mathbb{I}\left[t_j\leq t\right]\lambda(t-t_j), t_i\right)\right\}, \tag{19}$$

This is quite a realistic hypothesis. In fact, when an individual gets infected, its infectivity is not constant in time in real situations but it depends on the viral load of the infectious individuals. Typically, infectivity is very low in a first time-window after the contagion, then increases according to time scales that depend on the type of pathogens, and finally, it decreases to zero. In particular, the generative rate λ depends on the time elapsed since infection, i.e. $\lambda(t-t_j)$. This introduces a Non-Markovian character to the dynamic because in order to know the configuration at time $t+dt$ it is not sufficient to know the state at time t, but a memory of all the infection times of each individual must be kept. Even though the generative model becomes more complex with respect to the Markovian case described above, the inference with CVA has almost no modifications. For this reason, the same inferential parameters introduced in the Markovian case are used, with the same interpretation. The only difference is that the effective interaction rate is now defined as

$$\lambda_{ji}^{eff}(t|t_j) := \frac{\lambda(t-t_j)\lambda_i(t)}{\lambda_0} \tag{20}$$

(where λ_0 is a rate needed to preserve the correct dimension, but it can be numerically set to 1). It is worth noting that the effective infection rate encompasses both the time-dependent infectivity of individual j (which is given by the generative model) and the susceptibility of i to be infected (which instead is learned by CVA). The formula for the CVA ansatz is therefore

$$Q_\theta(\boldsymbol{x}) = \prod_i \left\{ \gamma_i \delta\left(t_i\right) + (1-\gamma_i) \times \Lambda \left(\sum_{j\in\partial i} \mathbb{I}\left[t_j^I \le t\right] \lambda_{ji}^{eff}\left(t|t_j\right) + \omega_i\left(t\right), t_i \right) \right\}, \tag{21}$$

.

SEIR model – To cope with the SEIR model, it suffices to slightly modify the formalism introduced for the SI models described above. Together with the infection rates and the source probabilities, it is necessary to include, in the generative model, the latency rate ν and the recovery rate μ, associated with the transitions $E \to I$ and $I \to R$, respectively. Hence, the parameters θ now encompass, for each individual i, the zero-patient probability, an infection rate function $\lambda_i(t)$, an auto-infection rate $\omega_i(t)$, and the recovery and latency rates $\mu_i(t)$ and $\nu_i(t)$. In the results shown in Section 3 of the main text, all these rates are parametrized using Gaussian functions, as in Eq. (17).
As a final remark, it is worth noting that, for both the SI and the SEIR models, the total number of parameters used by the CVA scales with N (in particular, their number is $7N$ for the SI and $13N$ for the SEIR).

S IV. SAMPLING IN THE CAUSAL VARIATIONAL APPROACH

Sampling from the posterior distribution is a difficult task in general. The CVA allows one to approximate the posterior with a distribution Q_θ from which, instead, it is possible to sample efficiently. The sampling process is crucial for performing the gradient descent described in Appendix S V. The present Appendix provides all the implementation details required to sample the probabilistic models considered in the paper.

Random walk – In Appendix S III, the ansatz Q takes the form of an inhomogeneous time-dependent random walk. suppose a trajectory $x = (x^0, x^1, \ldots, x^T)$ has to be drawn from Q, where x^t is the position of the walker at time t and $x^0 = 0$. To sample x, it is sufficient to start from state x^0 and repeat, up to the final time T, the temporal update $x^{t+1} = x^t + \Delta x$, where $\Delta x \in \{-1, +1\}$ is a binary random variable. The probability $r^t_{x^t}$, that $\Delta x = 1$, is the probability for a walker to jump to the right at time t starting from position x^t.

SI model – The parametrization used to infer the conditioned SI model is an SI model itself with self-infection probability, as described in Appendix S III. A Gillespie algorithm, described in detail in Algorithm 1, is used to sample from the SI model distribution. The idea behind the algorithm is to store in a queue $L = (L_1, L_2, \ldots, L_N)$ the infection times L_i of all the individuals. To this end, the first step consists of sampling all the zero-patient(s), i.e. all individuals i such that $L_i = 0$. Secondly, self-infection events are sampled for each individual i which is not a zero patient, with a random variable t_i extracted from the self-infection distribution $\omega_i(t)$.

Finally, contagion events are sampled by extracting from the queue the individual having the minimum value of L. If an individual i tries to infect another individual j at time t_{ij}, then j updates its infection time by taking the minimum between t_{ij} and its current value of L_j. In this way, the list is updated recursively.
As a final remark, notice that to ensure a proper epidemic process to take place, at least one zero patient is needed. Therefore, it is necessary to sample events with $L_i = 0$ by constraining a positive number n_{zp} of zero-patients. This procedure can be easily carried out recursively. For instance, starting from node 1, we set it to be a patient-zero with a probability

$$\mathbb{P}(L_1 = 0 | n_{zp} > 0) = \frac{\mathbb{P}(L_1 = 0, n_{zp} > 0)}{\mathbb{P}\left(n_{zp} > 0\right)} = \frac{\mathbb{P}(L_1 = 0)}{\mathbb{P}\left(n_{zp} > 0\right)} = \frac{\gamma_1}{1 - \prod_{i=1}^{N}\left(1 - \gamma_i\right)} \tag{22}$$

Then, the next node (say number 2) is set to be infected at $t = 0$ with a probability depending on L_1, namely:

$$\mathbb{P}(L_2 = 0 | L_1, n_{zp} > 0) = \begin{cases} \gamma_2 & \text{if } L_1 = 0 \\ \gamma_2 / \left(1 - \prod_{i \geq 2}(1 - \gamma_i)\right) & \text{if } L_1 > 0 \end{cases} \tag{23}$$

and the above strategy is iterated for every L_k. Intuitively, from Eq. (23) it follows that as soon as one zero patient is sampled, the remaining individuals' state is extracted independently with probability γ_i.

Algorithm 1 Sampling the effective (Non-Markovian) SI model

- Initialize a queue L
- **Loop** over population: $i : 1, \ldots, N$
  - $L[i] \leftarrow 0$ with probability: $\gamma_i / \left(1 - \prod_{j \geq i}(1 - \gamma_j)\right)$
  - **if** $L[i] = 0$ **break the loop**
- **Loop** over the remaining population:
  - $L[i] \leftarrow 0$ with probability γ_i
- **Loop** over the non zero-patients (variable of the loop: i)
  - $L[i] \leftarrow t_i$ where t_i is extracted from the self-infection rate ω_i
- **Loop** over the queue L entering by infection time (from the smallest to the highest)
  - Call i the element extracted from L
  - **Loop** over $j \in \partial i$
    * Extract the time t_{ij} at which i tries to infect j by extracting from the Gaussian distribution of λ_{ij}^{eff}
    * $L[j] \leftarrow \min\{L[j], t_{ij}\}$
  - Throw away i from the queue L and save $L[i]$ as the infection time of i.
- **Return** the list of infection times.

SEIR model – For the SEIR model, the sampling procedure is a straightforward generalization of the one just discussed. The only difference is that the queue contains all the transition time triplets t_i^E, t_i^I, t_i^R for each individual.
Remark. Notice that, since each sample is independent to the other, sampling can be performed in parallel. This implies, as shown in the next section, that CVA itself is a parallelizable algorithm.

S V. THE CAUSAL VARIATIONAL APPROACH: GRADIENT DESCENT

In the Causal Variational Approach, the parameters θ of the approximation are determined by minimizing the following KL divergence:

$$D_{KL}(Q_\theta||\mathbb{P}\left[\boldsymbol{x}|\mathcal{O}\right]) = \int d\boldsymbol{x}\, Q_\theta\left(\boldsymbol{x}\right)\log\left(\frac{Q_\theta(\boldsymbol{x})}{\mathbb{P}\left[\boldsymbol{x}|\mathcal{O}\right]}\right), \tag{24}$$

where Q_θ denotes the Causality ansatz. The integration is formal. If $\mathbb{P}\left[\boldsymbol{x}|\mathcal{O}\right]$ is continuous, then it corresponds to a Lebesgue integral, otherwise, it is a sum over the discrete state $\boldsymbol{x}$.

Gradient Descent – The KL divergence can be minimized by performing a gradient descent in the θ-parameter space. The parameters at iteration $k+1$ read

$$\theta^{(k+1)} = \theta^{(k)} - \epsilon\nabla_\theta D_{KL}(Q_\theta||\mathbb{P}\left[\boldsymbol{x}|\mathcal{O}\right]) \tag{25}$$

where ϵ is the learning rate. Before entering the calculation of the gradient, however, let us observe that, in general, θ contains parameters which have different scales, and, therefore, a straightforward implementation of eq. (25) may be inefficient. To solve this issue, we resort to a sign descender method [1], a simple scale-free descender. Briefly, it consists in moving in the direction of the partial derivative with respect to each parameter without using the information of the gradient's magnitude.

Finally, the update rule for a parameter θ_i used in this work has the following expression:

$$\theta_i^{(k+1)} = \theta_i^{(k)}\left[1 - \varepsilon\,\mathrm{sign}\left(\partial_{\theta_i} D_{KL}(Q_\theta||\mathbb{P}\left[\boldsymbol{x}|\mathcal{O}\right])\right)\right] \tag{26}$$

It is now necessary to evaluate the derivatives of $D_{KL}(Q_\theta||\mathbb{P}\left[\boldsymbol{x}|\mathcal{O}\right])$,

$$\begin{aligned}
\partial_{\theta_i} D_{KL}(Q_\theta||\mathbb{P}\left[\boldsymbol{x}|\mathcal{O}\right]) &= \partial_{\theta_i}\int d\boldsymbol{x} Q_\theta(x)\log\frac{Q_\theta(\boldsymbol{x})}{\mathbb{P}\left[\boldsymbol{x}|\mathcal{O}\right]} = \\
&= \partial_{\theta_i}\int d\boldsymbol{x} Q_\theta(\boldsymbol{x})\log\frac{Q_\theta(\boldsymbol{x})\mathbb{P}[\mathcal{O}]}{\mathbb{P}[\boldsymbol{x}]\mathbb{P}\left[\mathcal{O}|\boldsymbol{x}\right]} = \\
&= \int d\boldsymbol{x}\partial_{\theta_i}\left(Q_\theta(\boldsymbol{x})\log\frac{Q_\theta(\boldsymbol{x})}{\mathbb{P}\left[\boldsymbol{x}\right]\mathbb{P}\left[\mathcal{O}|\boldsymbol{x}\right]}\right) + \log\mathbb{P}\left[\mathcal{O}\right]\partial_{\theta_i}\int d\boldsymbol{x}\left(Q_\theta(\boldsymbol{x})\right) = \\
&= \int d\boldsymbol{x}\partial_{\theta_i}\left(Q_\theta(\boldsymbol{x})\log\frac{Q_\theta(\boldsymbol{x})}{\mathbb{P}\left[\boldsymbol{x}\right]\mathbb{P}\left[\mathcal{O}|\boldsymbol{x}\right]}\right)
\end{aligned} \tag{27}$$

in which the dependency on $P\left[\mathcal{O}\right]$ was neglected. We observe that

$$\int d\boldsymbol{x}\,\partial_{\theta_i}\left(Q_\theta(\boldsymbol{x})\log Q_\theta(\boldsymbol{x})\right) = \int d\boldsymbol{x}\,\partial_{\theta_i}Q_\theta(\boldsymbol{x})\log Q_\theta(\boldsymbol{x}) + \int d\boldsymbol{x}\,Q_\theta(\boldsymbol{x})\partial_{\theta_i}\log Q_\theta(\boldsymbol{x}) \tag{28}$$

In the above expression, the second term of the r.h.s. is 0, since

$$\int d\boldsymbol{x}Q_\theta(\boldsymbol{x})\partial_{\theta_i}\log Q_\theta(\boldsymbol{x}) = \int d\boldsymbol{x}\partial_{\theta_i}Q_\theta(\boldsymbol{x}) = \partial_{\theta_i}\int d\boldsymbol{x}Q_\theta(\boldsymbol{x}) = 0, \tag{29}$$

therefore

$$\begin{aligned}
\int d\boldsymbol{x}\partial_{\theta_i}\left(Q_\theta(\boldsymbol{x})\log Q_\theta(\boldsymbol{x})\right) &= \int d\boldsymbol{x}\left(\partial_{\theta_i}Q_\theta(\boldsymbol{x})\right)\log Q_\theta(\boldsymbol{x}) \\
&= \int d\boldsymbol{x}\frac{Q_\theta(\boldsymbol{x})}{Q_\theta(\boldsymbol{x})}\left(\partial_{\theta_i}Q_\theta(\boldsymbol{x})\right)\log Q_\theta(\boldsymbol{x}) = \\
&= \int d\boldsymbol{x}\,Q_\theta(\boldsymbol{x})\left(\partial_{\theta_i}\log Q_\theta(\boldsymbol{x})\right)\log Q_\theta(\boldsymbol{x}) = \\
&= \left\langle\log Q_\theta(\boldsymbol{x})\partial_{\theta_i}\log Q_\theta(\boldsymbol{x})\right\rangle_{Q_\theta}
\end{aligned} \tag{30}$$

In conclusion, the derivative of the KL divergence reads

$$\partial_{\theta_i} D_{KL}(Q_\theta || \mathbb{P}[\boldsymbol{x}|\mathcal{O}]) = \left\langle \log \frac{Q_\theta(\boldsymbol{x})}{\mathbb{P}[\boldsymbol{x}]\,\mathbb{P}[\mathcal{O}|\boldsymbol{x}]} \partial_{\theta_i} \log Q_\theta(\boldsymbol{x}) \right\rangle_{Q_\theta} \tag{31}$$

The problem of calculating the derivative of the KL divergence has been reduced to calculate the derivative of the logarithm of the ansatz and then to average over the ansatz. In principle, one may need to calculate

$$\partial_{\theta_i} \log Q_\theta(\boldsymbol{x}) = \sum_{j=1}^{N} \partial_{\theta_i} \log q_j(x_j, x_{\partial j}) \tag{32}$$

but it is reasonable to assume that the dependency of each parameter θ_i occurs only on the term $\log q_i$. This hypothesis is true for all the examples and applications presented in this work, as described in the previous Appendix. Therefore

$$\partial_{\theta_i} D_{KL}(Q_\theta || \mathbb{P}[\boldsymbol{x}|\mathcal{O}]) = \left\langle \log \frac{Q_\theta(\boldsymbol{x})}{\mathbb{P}[\boldsymbol{x}]\,\mathbb{P}[\mathcal{O}|\boldsymbol{x}]} \partial_{\theta_i} \log q_i(x_i, x_{\partial i}) \right\rangle_{Q_\theta} \tag{33}$$

A last manipulation consists in subtracting the quantity $0 = \left\langle \log \frac{Q_\theta(\boldsymbol{x})}{\mathbb{P}[\boldsymbol{x}]\mathbb{P}[\mathcal{O}|\boldsymbol{x}]} \right\rangle_{Q_\theta} \langle \partial_{\theta_i} \log Q_\theta(\boldsymbol{x}) \rangle_{Q_\theta}$ to equation (33) in order to obtain:

$$\partial_{\theta_i} D_{KL}(Q_\theta || \mathbb{P}[\boldsymbol{x}|\mathcal{O}]) = \left\langle \left(\log \frac{Q_\theta(\boldsymbol{x})}{\mathbb{P}[\boldsymbol{x}]\,\mathbb{P}[\mathcal{O}|\boldsymbol{x}]} - \left\langle \log \frac{Q_\theta(\boldsymbol{x})}{\mathbb{P}[\boldsymbol{x}]\,\mathbb{P}[\mathcal{O}|\boldsymbol{x}]} \right\rangle_{Q_\theta} \right) \partial_{\theta_i} \log q_i(x_i, x_{\partial i}) \right\rangle_{Q_\theta} . \tag{34}$$

This manipulation is called *variance reduction* [2] and is known to facilitate the gradient descent. Averages are evaluated by sampling from Q_θ using the implementation details given in Appendix S IV. Since sampling can be performed in parallel, also the gradient computation is parallelizable, due to the form of equation (34). The learning process is therefore the following:

1. The parameters are initialized to θ^0 (we initialize them with the values of the generative model, where known. For example, in the random walk example the jump probabilities are all initialized to 1/2).

2. The derivatives of the KL divergence are performed by sampling from Q_{θ_0} and through Eq. (34).

3. The parameters are updated to θ^1 by using (26).

4. Steps 2. and 3. are repeated.

5. The process stops when $D_{KL}(Q_\theta || \mathbb{P}[\boldsymbol{x}|\mathcal{O}])$ does not significantly change after two consecutive iterations.

In the evaluation of the KL divergence (or its derivatives), it is necessary to evaluate quantities that include $\log \mathbb{P}(\mathcal{O} \mid \boldsymbol{x})$. When $\mathcal{O}$ imposes a hard constraint on the trajectory - as it happens in the epidemic models with noiseless observations - the violation of one of the constraints (which is likely to occur especially in the first stages of the gradient descent) would result in $\log \mathbb{P}(\mathcal{O} \mid \boldsymbol{x}) \to -\infty$. To avoid this issue, it is convenient to relax (soften) all the constraints: a small acceptance $p \sim 10^{-10}$ is introduced, in such a way that $\log \mathbb{P}(\mathcal{O} \mid \boldsymbol{x})$ has always non-diverging values. As a consequence, the distribution $Q_\theta(\boldsymbol{x})$ resulting from the KL minimization gives a non-zero (yet very small) probability to events $\boldsymbol{x}$ which do not satisfy the constraint.

S VI. INFERENCE OF HYPERPARAMETERS

As explained in Section 3 in the main text, the term *hyperparameters* refers to parameters of a generative model. For example, the Random Walk presented in Section 2 in the main text has a unique hyperparameter, namely the probability of a right jump, which is set to 1/2 for all times and sites. In the SI model, the hyperparameters are the zero-patient probability γ and the infection rate λ while in the SEIR model, we need to add to the hyperparameters set the latency rate ν and the recovery rate μ. Usually, in inference problems, they are not known as the only source of information, in fact, are the observations. However, inferring the hyperparameters from observations is crucial to ensure an effective solution to the underlying inference problem. The CVA allows one to estimate them. Let us call the set of hyperparameters θ^p. First, notice that in general, not only the generating distribution $\mathbb{P}[\boldsymbol{x}]$, but also the ansatz Q_{θ,θ^p} can depend on the hyperparameters θ^p. This dependency might be present when using part of the generative model in the ansatz distribution. The example implemented in this paper is the case of the Non-Markovian SI model (see Appendix S III). In that case, the infectivity function depends on both the inferred parameters ($\{\lambda_i\} \in \theta$) and the relative infectivity of the generative model ($\lambda \in \theta^p$), as shown in Eq. (20). To optimize the parameters, we will use a Maximum Likelihood (ML) approax, maximizing $\log \mathbb{P}[O|\theta^p]$ with respect to θ^p:

$$\begin{aligned}\arg\max_{\theta^p} \log \mathbb{P}[O|\theta^p] &\approx \arg\min_{\theta^p}\min_{\theta} \left[D_{KL}\left(Q_{\theta,\theta^p}||\mathbb{P}[\boldsymbol{x}|O,\theta^p]\right) - \log \mathbb{P}[O|\theta^p]\right] \\ &= \arg\min_{\theta^p}\min_{\theta} \int d\boldsymbol{x} Q_{\theta,\theta^p}[\boldsymbol{x}] \log \frac{Q_{\theta,\theta^p}[\boldsymbol{x}]}{\mathbb{P}[O|\boldsymbol{x},\theta^p]\,\mathbb{P}[\boldsymbol{x}|\theta^p]}\end{aligned}$$

where the $\approx$ becomes an equality if the Ansatz Q_{θ,θ^p} is general enough to represent $\mathbb{P}[\boldsymbol{x}|O,\theta^p]$. So our task is to minimize $\int d\boldsymbol{x} Q_{\theta,\theta^p}[\boldsymbol{x}] \log \frac{Q_{\theta,\theta^p}[\boldsymbol{x}]}{\mathbb{P}[O|\boldsymbol{x},\theta^p]\mathbb{P}[\boldsymbol{x}|\theta^p]}$ with respect to both θ and θ^p. In doing so, we will simultaneously find our best approximation Q_{θ,θ^p} of the posterior $\mathbb{P}[\boldsymbol{x}|O,\theta^p]$. Let us compute gradients:

$$\partial_\theta \int d\boldsymbol{x} Q_{\theta,\theta^p}[\boldsymbol{x}] \log \frac{Q_{\theta,\theta^p}[\boldsymbol{x}]}{\mathbb{P}[O|\boldsymbol{x},\theta^p]\,\mathbb{P}[\boldsymbol{x}|\theta^p]} = \partial_\theta \int d\boldsymbol{x} Q_{\theta,\theta^p}[\boldsymbol{x}] \log \frac{Q_{\theta,\theta^p}[\boldsymbol{x}]}{\mathbb{P}[O|\boldsymbol{x},\theta^p]\,\mathbb{P}[\boldsymbol{x}|\theta^p]} \tag{35}$$

$$\begin{aligned}&= \int d\boldsymbol{x} \partial_\theta Q_{\theta,\theta^p}[\boldsymbol{x}] \log \frac{Q_{\theta,\theta^p}[\boldsymbol{x}]}{\mathbb{P}[O|\boldsymbol{x},\theta^p]\,\mathbb{P}[\boldsymbol{x}|\theta^p]} + \\ &+ \int d\boldsymbol{x} Q_{\theta,\theta^p}[\boldsymbol{x}]\, \partial_\theta \log \frac{Q_{\theta,\theta^p}[\boldsymbol{x}]}{\mathbb{P}[O|\boldsymbol{x},\theta^p]\,\mathbb{P}[\boldsymbol{x}|\theta^p]}\end{aligned} \tag{36}$$

$$= \int d\boldsymbol{x} Q_{\theta,\theta^p}[\boldsymbol{x}]\, \partial_\theta \log Q_{\theta,\theta^p}[\boldsymbol{x}] \log \frac{Q_{\theta,\theta^p}[\boldsymbol{x}]}{\mathbb{P}[O|\boldsymbol{x},\theta^p]\,\mathbb{P}[\boldsymbol{x}|\theta^p]} \tag{37}$$

$$= \left\langle \partial_\theta \log Q_{\theta,\theta^p}[\boldsymbol{x}] \log \frac{Q_{\theta,\theta^p}[\boldsymbol{x}]}{\mathbb{P}[O|\boldsymbol{x},\theta^p]\,\mathbb{P}[\boldsymbol{x}|\theta^p]} \right\rangle_{Q_{\theta,\theta^p}} \tag{38}$$

where in Eq. 36 we used the fact that $\partial_\theta Q_{\theta,\theta^p} = Q_{\theta,\theta^p}\partial_\theta \log Q_{\theta,\theta^p}$ and that $\int d\boldsymbol{x} Q_{\theta,\theta^p}[\boldsymbol{x}]\,\partial_\theta \log Q_{\theta,\theta^p}[\boldsymbol{x}] \equiv 0$.

$$\partial_{\theta^p}\int d\boldsymbol{x} Q_{\theta,\theta^p}[\boldsymbol{x}]\log\frac{Q_{\theta,\theta^p}[\boldsymbol{x}]}{\mathbb{P}[O|\boldsymbol{x},\theta^p]\,\mathbb{P}[\boldsymbol{x}|\theta^p]} = \partial_{\theta^p}\int dx Q_{\theta,\theta^p}[\boldsymbol{x}]\log\frac{Q_{\theta,\theta^p}[\boldsymbol{x}]}{\mathbb{P}[O|\boldsymbol{x},\theta^p]\,\mathbb{P}[\boldsymbol{x}|\theta^p]} \tag{39}$$

$$\begin{aligned} &= \int d\boldsymbol{x}\partial_{\theta^p} Q_{\theta,\theta^p}[\boldsymbol{x}]\log\frac{Q_{\theta,\theta^p}[\boldsymbol{x}]}{\mathbb{P}[O|\boldsymbol{x},\theta^p]\,\mathbb{P}[\boldsymbol{x}|\theta^p]} + \\ &+ \int d\boldsymbol{x} Q_\theta[x]\,\partial_{\theta^p}\log\frac{Q_{\theta,\theta^p}[\boldsymbol{x}]}{\mathbb{P}[O|\boldsymbol{x},\theta^p]\,\mathbb{P}[\boldsymbol{x}|\theta^p]} \end{aligned} \tag{40}$$

$$\begin{aligned} &= \int dx Q_{\theta,\theta^p}[\boldsymbol{x}]\,\partial_{\theta^p}\log Q_\theta[\boldsymbol{x}]\log\frac{Q_{\theta,\theta^p}[\boldsymbol{x}]}{\mathbb{P}[O|\boldsymbol{x},\theta^p]\,\mathbb{P}[\boldsymbol{x}|\theta^p]} + \\ &- \int dx Q_{\theta,\theta^p}[\boldsymbol{x}]\,\partial_{\theta^p}\log\mathbb{P}[O|\boldsymbol{x},\theta^p]\,\mathbb{P}[\boldsymbol{x}|\theta^p] \end{aligned} \tag{41}$$

$$\begin{aligned} &= \left\langle \partial_{\theta^p}\log Q_{\theta,\theta^p}[\boldsymbol{x}]\log\frac{Q_{\theta,\theta^p}[\boldsymbol{x}]}{\mathbb{P}[O|\boldsymbol{x},\theta^p]\,\mathbb{P}[\boldsymbol{x}|\theta^p]}\right\rangle_{Q_{\theta,\theta^p}} + \\ &- \left\langle \partial_{\theta^p}\log\mathbb{P}[O|\boldsymbol{x},\theta^p]\,\mathbb{P}[\boldsymbol{x}|\theta^p]\right\rangle_{Q_{\theta,\theta^p}} \end{aligned} \tag{42}$$

Unsurprisingly, Eq. (38) is identical to Eq. (31). Eq. (42) is similar to Eq. (31), with an extra second term that accounts for the direct dependence of the model on the hyperparameters. Note that this second term can be interpreted as a negated derivative of the energy in an Expectation Maximization (EM) scheme. The learning process for θ^p is analogous to learning θ: the averages in r.h.s of Eq. (42) are calculated by sampling and then the hyperparameters are updated using the Sign Descender rule. From a computational point of view, it is convenient to update both the parameters θ and the hyperparameters θ^p at each iteration.

S VII. OTHER INFERENTIAL METHODS

This section provides a brief description of the other inferential techniques whose performances are compared with those of Causal Variational Approach in Section 3 in the main text.

Sib – This method is based on a Belief Propagation approach to epidemic spreading processes, that allows one to compute, in an efficient and distributed way, the marginals over a posterior distribution (Eq. (1)) for compartmental epidemic models with non-recurrent dynamics (eventually non-Markovian). It shows very good performances when epidemic models take place on random contact networks, while it may suffer from the presence of loops in the graph. A detailed explanation of this method can be found in [3].

Mean Field (MF) – This method is based on a heuristic way to deal with observations from clinical tests and on a Mean Field approximation of the prior distribution, which is considered to be factorized over nodes at each time. An advantage of this method relies on its simplicity and small computational cost; however, it typically shows poorer performances with respect to the other methods. We refer to [3] for additional details about the MF approximation. The heuristic scheme is instead discussed in the next paragraph.

Heuristic (heu) – The MF method developed in [3] and described above relies on two approximations. The first is a heuristic way to deal with observations: it consists in assuming that if an individual has tested positive at time t, then it became positive at time $t-\tau$, with τ properly tuned (to further details we refer to [3]). The second is an MF ansatz for the prior distribution. It is natural to ask whether such a heuristic is a good approximation for the observation constraints, regardless of the MF factorization ansatz. We, therefore, replace the MF estimation of marginal probabilities by sampling trajectories forward in time. As shown in Section 3, this method shows slightly better performances w.r.t. MF. The heuristic, therefore, is a good guess for epidemic risk assessment. As a final remark, the Causal Variational Approach can actually be considered as a further extension of the MF approximation [3] in the following sense:

1. it gives a full justification to the heuristic using a variational principle;

2. it extends [3] by allowing the patient zero inference;

3. it allows one to infer the hyperparameters of the generative model and to perform epidemic reconstruction in the case of non-Markovian dynamics.

Monte Carlo The results obtained for the SI model in Figs. 2-3 in the main text are also compared to a standard Markov-Chain Monte-Carlo (MCMC) sampling for the posterior distribution. Since epidemic trajectories can be fully described in terms of the infection time vector $\boldsymbol{t} = (t_1, \ldots, t_N)$, the Markov chain defines dynamics on these continuous variables that eventually converge to a stationary distribution (i.e. the posterior). At each step of the MCMC, a node i is randomly selected and a new value of its infection time - denoted with t'_i - is proposed, by drawing it from a suitable kernel $\mathcal{K}(t'_i \mid t_i)$. We adopted a Gaussian kernel centered at t_i, with fixed standard deviation δ. The proposed value t'_i sampled from $\mathcal{K}$ is then clamped within the interval $[0,T]$: this procedure allows to sample with non-zero probability the two extreme values, associated to node i being a patient-zero (when $t_i = 0$), or node i never being infected (in which case its infection time is formally set to infinity, as previously discussed). This procedure is equivalent to use as proposal kernel a 2-sided rectified Gaussian distribution in the window $[0,T]$. The acceptance probability of such a proposal is computed as

$$p_{\text{acc}} = \min\left(1, \frac{\mathbb{P}[\boldsymbol{t}'_i|\mathcal{O}]}{\mathbb{P}[\boldsymbol{t}|\mathcal{O}]} \frac{\tilde{\mathcal{K}}(t_i \mid t'_i, \delta)}{\tilde{\mathcal{K}}(t'_i \mid t_i, \delta)}\right) \tag{43}$$

where $\boldsymbol{t}'_i$ represents an epidemic trajectory where only t_i is modified to the corresponding proposed value, namely $\boldsymbol{t}'_i = (t_1, \ldots, t'_i, \ldots, t_N)$, and $\tilde{\mathcal{K}}$ is the rectified kernel given by:

$$\tilde{\mathcal{K}}(x \mid t_i, \delta) = \Phi(0; t_i, \delta)\, \delta(x) + \mathbb{I}[x \in (0,T)]\, K(x; t_i, \delta) + [1 - \Phi(T; t_i, \delta)]\, \delta(x - T) \tag{44}$$

with $K(x; \mu, \sigma)$ being the probability density of a Gaussian random variable and $\Phi(x; \mu, \sigma)$ its cumulative function. The initial condition for the Markov Chain is sampled from the prior distribution of the SI model. In order to have a fair comparison, the number of samples collected during the MCMC is equal to the one used by CVA. To diminish the effect of initial equilibration time an initial number of steps is typically required to let the MC forget the initial condition and sample efficiently the posterior distribution; notice that a "step" consists in proposing a move for each node in a random permutation.

Soft-Margin – The Soft-Margin estimator is described in [4]. We adapted this method by sampling from the prior probability distribution $\mathbb{P}[\boldsymbol{x}]$ and weighting each sample with the observation likelihood $\mathbb{P}[\mathcal{O}|\boldsymbol{x}]$, in which we introduced a small artificial noise in the form of a false rate, which softened the constraints, improving the performances of the method. The technique is asymptotically exact. However, when the population size grows, the probability to sample a trajectory $\boldsymbol{x}$ which satisfies the observation constraints $\mathbb{P}[\mathcal{O}|\boldsymbol{x}]$ dramatically decreases. Therefore, we expect the method to be quite slow for large population sizes in order to well-approximate the posterior distribution.

S VIII. MODELS OF CONTACT NETWORKS USED IN THE NUMERICAL SIMULATIONS

Proximity model – The proximity contact graph is obtained by distributing N individuals uniformly in a square of side $\sqrt{N}$ and generating the contacts as follows. A contact can be established between two individuals i and j with a probability $e^{-d_{ij}/\ell}$, where d_{ij} is the Euclidean distance between the points i and j are located and ℓ is a length scale that can be tuned to change the density of the contact graph.

OpenABM-Covid19 – A synthetic contact network has been generated through OpenABM, a platform used to set up realistic epidemic instances on dynamic contact networks where a set of intervention measures have been applied and studied in Ref. [5]. The underline contact pattern is a superposition of two static graphs, one a complete graph representing interaction within households and a second small-word network mirroring occupation relationships. A random time-varying network is instead used and rebuilt on a daily basis to model contacts in public transportation, transient social gatherings, etc. The number of interactions through the random network

is extracted from a negative binomial distribution to allow for rare super-spreading events. Memberships to both fixed and dynamic graphs are determined by the age of individuals, i.e. children live with adults, elderly people have fewer interactions than other age groups, etc.
In this work, we generated an instance of $N = 1000$ individuals using default parameters.

Spatio-temporal model from geolocation data – Dynamic contact network instances with realistic patterns of time-dependent contacts can be generated using the spatio-temporal model in Ref. [6]. According to this model, individuals are assigned to households that are localized in an urban area according to the actual population density. According to available geo-location data, other venues (schools and research institutes, social places, bus stops, workplaces, and supermarkets) are similarly displaced in the map. In a mobility simulation, individuals can visit a number of locations with a probability that decreases as the household-target distance increases. The duration of contacts between individuals concurrently visiting the same venue is assumed to be known and gathered by contact tracing smartphone applications. Some interesting and realistic features naturally arise from this contact dynamics, such as the presence of super-spreaders, e.g. the number of infections caused by infectious individuals is over-dispersed. In the present work, only a small example of such kind of dynamically generated contact network was analyzed. The contact-network instances used to obtain results in Fig. 3 in the main text represent the interaction graph among a small community of $N = 904$ individuals moving on a coarse-grained version of the city of Tübingen, in Germany. Due to the rescaling of the population, also the number of venues in the urban area was rescaled by a factor of 20.

S IX. PROOF THAT CONDITIONING A MARKOV PROCESS WITH LOCAL TIME OBSERVATIONS LEADS TO A MARKOV PROCESS

Let us suppose to have a Markov process described by the following probability distribution:

$$\mathbb{P}[\mathbf{x}] = \mathbb{P}[x(0)] \prod_{t=1}^{T-1} \mathbb{P}[x(t)|x(t-1)] \tag{45}$$

Now we constrain the dynamics to a set $\mathcal{O} = ((O_1, t_1) \dots, (O_M, t_M))$ of local observations in time, i.e. with probability law $\mathbb{P}[\mathcal{O}|x(0), \dots, x(T)] = \prod_t \prod_{\mu:t_\mu=t} \mathbb{P}[O_\mu|x(t)] =: \prod_t \mathbb{P}[O_t|x(t)]$. We will show that the posterior distribution is still a Markov process, i.e.

$$\mathbb{P}[x(0), \dots, x(T)|\mathcal{O}] = \mathbb{P}[x(0)|\mathcal{O}] \prod_{t=1}^{T} \mathbb{P}[x(t)|x(t-1), \mathcal{O}] \tag{46}$$

The posterior probability can be rewritten using Bayes' Theorem as:

$$\mathbb{P}[\mathbf{x}|\mathcal{O}] \propto \mathbb{P}[\mathbf{x}]\mathbb{P}[\mathcal{O}|\mathbf{x}] = \mathbb{P}[x(0)] \left(\prod_{t=0}^{T-1} \mathbb{P}[x(t+1)|x(t)] \right) \left(\prod_{t=0}^{T} \mathbb{P}[\mathcal{O}_t|x(t)] \right), \tag{47}$$

where we exploited that the observations are local in time and therefore the conditional probability factorizes. From the above equation we can try to calculate the posterior probability at a given time s conditioned to the

whole past:

$$\begin{aligned}
\mathbb{P}[x(s)|x(0),x(1),\dots,x(s-1),\mathcal{O}] &= \frac{\mathbb{P}[x(s),x(0),x(1),\dots,x(s-1)|\mathcal{O}]}{\mathbb{P}[x(0),x(1),\dots,x(s-1)|\mathcal{O}]} \\
&\propto \mathbb{P}[x(s),x(0),x(1),\dots,x(s-1)|\mathcal{O}] \\
&= \sum_{x(s+1),\dots,x(T)} \mathbb{P}[x(0),\dots,x(T)|\mathcal{O}] \\
&= \mathbb{P}[x(0)] \left(\prod_{r=0}^{s-2} \mathbb{P}[x(r+1)|x(r)]\right) \left(\prod_{r=0}^{s-1} \mathbb{P}[\mathcal{O}_r|x_r]\right) f\left(x(s),x(s-1),\mathcal{O}\right) \\
&\propto f(x(s),x(s-1),\mathcal{O}).
\end{aligned} \tag{48}$$

where the symbol $\propto$ is intended with respect to x_s so all terms that do not depend on x_s can be removed and we defined:

$$f(x(s),x(s-1),\mathcal{O}) := \mathbb{P}[x(s)|x(s-1),\mathcal{O}] \sum_{x(s+1),\dots,x(T)} \left(\prod_{r=s}^{T-1} \mathbb{P}[x(r+1)|x(r)]\right) \left(\prod_{r=s}^{T} \mathbb{P}[\mathcal{O}_r|x(r)]\right), \tag{49}$$

Now:

$$\mathbb{P}[x(s)|x(s-1),\mathcal{O}] = \sum_{x(s-2),\dots,x(0)} \mathbb{P}[x(s)|x(s-1),\dots,x(0),\mathcal{O}]\mathbb{P}[x(s-1),\dots,x(0)|\mathcal{O})] \tag{50}$$

$$\propto f(x(s),x(s-1),\mathcal{O}) \sum_{x(s-2),\dots,x(0)} \mathbb{P}[x(s-1),\dots,x(0)|\mathcal{O}] \tag{51}$$

$$\propto f(x(s),x(s-1),\mathcal{O}) \tag{52}$$

$$\propto \mathbb{P}[x(s)|x(s-1),\dots,x(0),\mathcal{O}] \tag{53}$$

Where again, any term that is constant with respect to x_s was removed. As $\mathbb{P}[x(s)|x(s-1),\mathcal{O}] \propto \mathbb{P}[x(s)|x(s-1),\dots,x(0),\mathcal{O}]$ and both are normalized with respect to x_s, they must be identical. Therefore, using equation (4) we conclude the proof. We can conclude that, at fixed observations, the posterior distribution at a given time s can be represented as a Markov process, i.e. depending only on the previous time $s-1$. However, the transition probabilities between these two times will depend on the observations at future times, i.e. $t \geq s$ and a closed formula would require to recast $f\left(x(s),x(s-1) \mid \mathcal{O}\right) = \tilde{\mathbb{P}}[x(s)|x(s-1),\mathcal{O}]$, which involves an exponential number of terms to be computed (remember that x is the full state of all the degrees of freedom). In this perspective, the CVA further assumes that even these transition probabilities factorize, i.e. there is a spatial conditional independence over the posterior, a necessary ingredient for sampling efficiency.

---

[1] Jeremy Bernstein, Yu-Xiang Wang, Kamyar Azizzadenesheli, and Animashree Anandkumar. signSGD: Compressed Optimisation for Non-Convex Problems. In *Proceedings of the 35th International Conference on Machine Learning*, pages 560–569. PMLR, July 2018. ISSN: 2640-3498.

[2] Dian Wu, Lei Wang, and Pan Zhang. Solving statistical mechanics using variational autoregressive networks. *Phys. Rev. Lett.*, 122:080602, Feb 2019.

[3] Antoine Baker, Indaco Biazzo, Alfredo Braunstein, Giovanni Catania, Luca Dall'Asta, Alessandro Ingrosso, Florent Krzakala, Fabio Mazza, Marc Mézard, Anna Paola Muntoni, Maria Refinetti, Stefano Sarao Mannelli, and Lenka Zdeborová. Epidemic mitigation by statistical inference from contact tracing data. *Proceedings of the National Academy of Sciences*, 118(32):e2106548118, August 2021. Publisher: Proceedings of the National Academy of Sciences.

[4] Nino Antulov-Fantulin, Alen Lančić, Tomislav Šmuc, Hrvoje Štefančić, and Mile Šikić. Identification of patient zero in static and temporal networks: Robustness and limitations. *Phys. Rev. Lett.*, 114:248701, Jun 2015.

[5] Robert Hinch, William J. M. Probert, Anel Nurtay, Michelle Kendall, Chris Wymant, Matthew Hall, Katrina Lythgoe, Ana Bulas Cruz, Lele Zhao, Andrea Stewart, Luca Ferretti, Daniel Montero, James Warren, Nicole Mather, Matthew Abueg, Neo Wu, Olivier Legat, Katie Bentley, Thomas Mead, Kelvin Van-Vuuren, Dylan Feldner-Busztin, Tommaso Ristori, Anthony Finkelstein, David G. Bonsall, Lucie Abeler-Dörner, and Christophe Fraser. OpenABM-covid19—an agent-based model for non-pharmaceutical interventions against COVID-19 including contact tracing. *PLOS Computational Biology*, 17(7):e1009146, 2021. Publisher: Public Library of Science.

[6] Lars Lorch, Heiner Kremer, William Trouleau, Stratis Tsirtsis, Aron Szanto, Bernhard Schölkopf, and Manuel Gomez-Rodriguez. Quantifying the effects of contact tracing, testing, and containment measures in the presence of infection hotspots. *ACM Transactions on Spatial Algorithms and Systems*, 2022.
